# Genome-wide identification of Kanamycin B binding RNA in *Escherichia coli*

**DOI:** 10.1186/s12864-023-09234-3

**Published:** 2023-03-16

**Authors:** Yaowen Chang, Wenxia Sun, Alastair I. H. Murchie, Dongrong Chen

**Affiliations:** 1 Fudan University Pudong Medical Center, and Institute of Biomedical Sciences, Shanghai Medical College, Key Laboratory of Medical Epigenetics and Metabolism, Fudan University, 200032 Shanghai, China; 2 Key Laboratory of Metabolism and Molecular Medicine, Ministry of Education, School of Basic Medical Sciences, Fudan University, Shanghai, 200032 China

**Keywords:** Kanamycin B, Pull-down assay, Genome-wide aminoglycoside RNA interactions, RNA-seq

## Abstract

**Background:**

The aminoglycosides are established antibiotics that inhibit bacterial protein synthesis by binding to ribosomal RNA. Additional non-antibiotic aminoglycoside cellular functions have also been identified through aminoglycoside interactions with cellular RNAs. The full extent, however, of genome-wide aminoglycoside RNA interactions in *Escherichia coli* has not been determined. Here, we report genome-wide identification and verification of the aminoglycoside Kanamycin B binding to *Escherichia coli* RNAs. Immobilized Kanamycin B beads in pull-down assays were used for transcriptome-profiling analysis (RNA-seq).

**Results:**

Over two hundred Kanamycin B binding RNAs were identified. Functional classification analysis of the RNA sequence related genes revealed a wide range of cellular functions. Small RNA fragments (ncRNA, tRNA and rRNA) or small mRNA was used to verify the binding with Kanamycin B in vitro. Kanamycin B and ibsC mRNA was analysed by chemical probing.

**Conclusions:**

The results will provide biochemical evidence and understanding of potential extra-antibiotic cellular functions of aminoglycosides in *Escherichia coli.*

**Supplementary Information:**

The online version contains supplementary material available at 10.1186/s12864-023-09234-3.

## Introduction

The aminoglycosides are effective broad-spectrum antibiotics that primarily inhibit protein synthesis by binding to the 30S ribosomal subunit at the A site in the decoding region of 16SrRNA leading to inhibition of translocation and mistranslation of mRNA [1–3]. Accumulating evidence confirms that, in addition to their function as bactericidal antibiotics, the aminoglycosides also have additional broader and diverse cellular roles. As natural products, the aminoglycosides are synthesized by complex and tightly regulated biosynthetic pathways [4–6]. The producer organisms, typically *Actinomycetes*, encode resistance factors that encompass all of the major mechanisms that confer antibiotic resistance to self-protect the producer form the antibiotic. These include target site modification (ribosomal methyltransferases), drug modification (acetyl, adenyl and phosphotransferases) and efflux pumps [7]. These resistance mechanisms are recruited to protect antibiotic resistant pathogens, where exposure to low levels of the aminoglycosides induces resistance in the clinic [8–10]. Aminoglycosides have been shown to bind to the 5’ leader RNA of aminoglycoside acetyltransferase (AAC) or adenyltransferase (AAD) causes a structural transition around the ribosome binding site which in turn regulates the translation of these resistance genes [11–14]. Aminoglycoside binding sites are known in rRNA [3, 15], HIV trans-activating region [16] and Rev responsive element [17] and autocatalytic ribozymes [18]. Aminoglycosides are known to inhibit hammerhead ribozyme cleavage [19] and can enhance hairpin ribozyme activity in the absence of metal ions [20]. The aminoglycosides have been shown to modulate the activity of the nucleolytic ribozymes [19–23]. Aminoglycoside antibiotics can induce bacterial biofilm formation in *Pseudomonas aeruginosa* and in *Escherichia coli* (*E.coli*) [24]. In *E.coli* the transcriptional SOS stress response is induced directly by the fluoroquinolones [25], Beta-lactams [26] and trimethoprim [27] antibiotics and indirectly through the induction of SOS regulated DNA polymerases by the tetracycline antibiotics [28]. Although the SOS response is triggered by the antibiotics in a number of pathogens including *Vibrio cholerae*, the aminoglycosides do not trigger the SOS response in *E.coli* [29, 30].

The development of in vitro selection methods, systematic evolution of ligands by exponential enrichment (SELEX) allows the selection of RNA aptamer domain for a specific small molecule [31–34]. SELEX enables the identification of RNA aptamers that bind small ligands with high affinity [35–38]. RNA aptamers have been identified using SELEX against a variety of small molecules such as Theophylline or antibiotics including tetracycline [39], ciprofloxacin [40] and various aminoglycosides. Identification of RNA aptamers against aminoglycosides such as lividomycin [41], neomycin [42], tobramycin [43, 44] and Kanamycin B in vitro has been reported. In particular, a stem loop structural motif that binds to Kanamycin B has been identified through SELEX. Although the sequences of the Kanamycin B binding motifs were completely different from the known sequences of the aminoglycoside-binding domains, the consensus RNA motifs share a shape-specific bulged stem loop that binds the aminoglycosides with nanomolar affinity [45].

Although a variety of non-antibiotic cellular functions of aminoglycosides have been described, and aminoglycoside binding aptamers have been identified by SELEX, few studies that investigate cellular RNAs that bind Kanamycin B have been reported so far. Such cellular RNAs that bind to Kanamycin B may be potential functional sites for Kanamycin B that reveal further novel cellular effects of Kanamycin B. Here we report the genome-wide identification of Kanamycin B binding to RNA in *E. coli* by using immobilized Kanamycin B beads and transcriptome-profiling analysis (RNA-seq). We identified over two hundred RNAs that bind to Kanamycin B through the Kanamycin B pull-down assay. Functional classification analysis was performed on the RNA sequence related genes. We further verified the binding of RNA with Kanamycin B in vitro. We performed chemical probing of ibsC mRNA with Kanamycin B. Confirmation of cellular RNA binding by Kanamycin B is consistent with the notion that Kanamycin B might participate in wider biological processes or mimic an existing interactions, in addition to their function as ribosomal antibiotics.

## Results

### Overview of RNA-seq profiling and selection of RNA

In order to investigate potential cellular functions for Kanamycin B and identify Kanamycin B binding cellular RNAs, immobilized Kanamycin B Sepharose beads were used to select against total *E. coli* RNA, followed by transcriptome-profiling analysis (RNA-seq). Previous studies suggested that addition of 1 μM Kanamycin B had no effect on *E. coli* cell growth and was thus a reasonable concentration of Kanamycin B to use [46]. Cells were grown in the absence or presence of 1 μM Kanamycin B for 6 h and total RNA was extracted. The total RNA of the above two groups was pulled down with Kanamycin B Sepharose beads respectively, after washing the Kanamycin B binding RNA was subjected to RNA-seq analysis. The total RNA sample of each group was used as a control in parallel. Totally 12 stand-specific cDNA libraries were constructed containing 456.69million raw reads; 456.59 million clean reads (accounting for 99.98% of raw reads) were recorded after the removal of adapter sequences, low quality reads and those with more than 5% N bases. The clean reads with an average of ~ 97.68% Q30 (sequencing error rate < 0.1%) base rate for each sample, were used for further expression analysis. Ultimately, the high-quality clean reads were mapped to the *E. coli* str. K-12 substr. MG1655 genome.

**Fig. 1:**
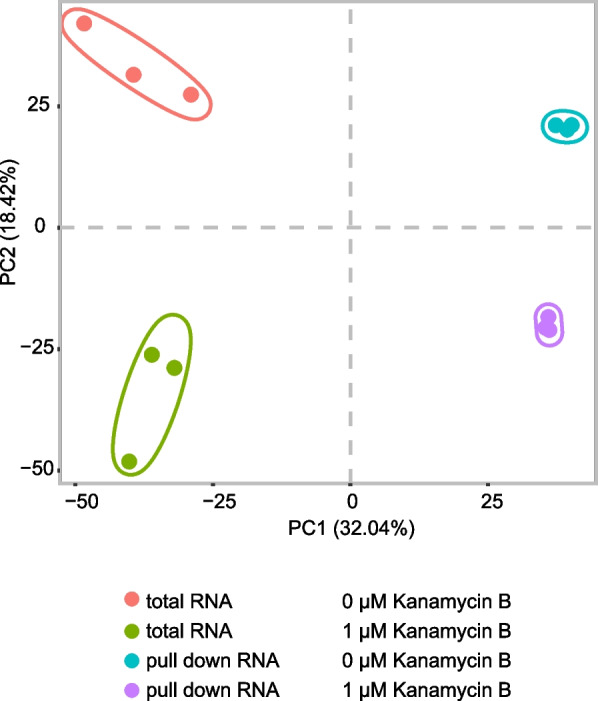
Principal component analysis of the RNA-Seq data. The small icon indicates the original samples, colours are used to differentiate the four treatment groups

To investigate the reproducibility of the three biological repeat experiments, principal component analysis (PCA) of the RNA-Seq data was used. The result showed that PC1 and PC2 had values of 32.04% and 18.42%, respectively, and accounted for 50.46% of the principal components. The PCA revealed consistency between the three biological replicates of total RNA or Kanamycin B binding RNA from samples without or with 1 μM Kanamycin B (Fig. 1). This analysis suggests reasonable reproducible of biological repeats (Fig. 1).

### Identification of Kanamycin B binding RNA

**Fig. 2:**
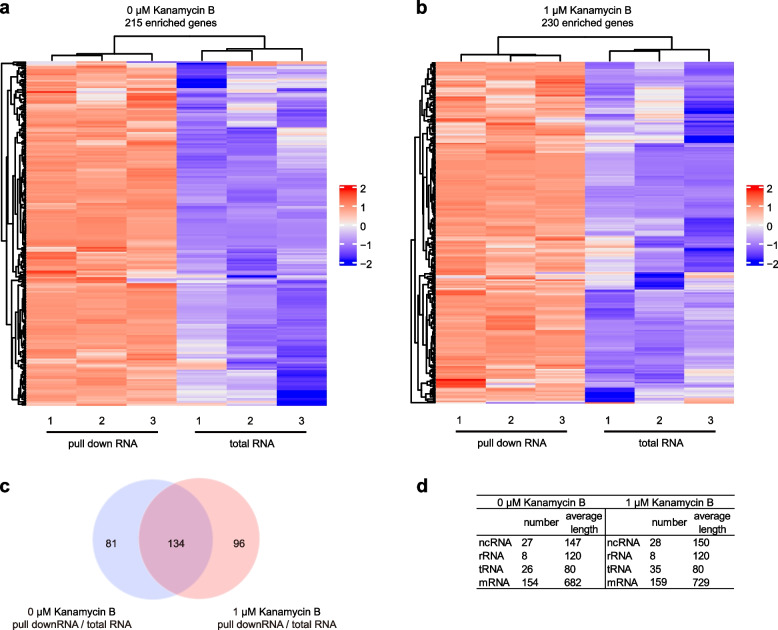
Analysis of enriched genes. **a** The hierarchical cluster analysis of enriched genes in 0 μM Kanamycin B pull down assay. **b** The hierarchical cluster analysis of enriched genes in 1 μM Kanamycin B pull down assay. Rows represent relative expression level of enriched genes and columns represent the different treatments. The relative quantitative changes within the row are shown in colour: red indicates a relative higher expression level whereas blue indicates a relative lower expression level. **c** The Venn diagram displays the number of the shared and unique genes that are enriched in the Kanamycin B pull down assay. **d** Tabulation of the classification of the Kanamycin B pull down RNA

To identify the Kanamycin B binding RNA from the RNA-Seq data, DEseq2 software was used to select RNA sequences that are enriched more than twofold and p value less than 0.05 in the Kanamycin B pull down RNAs compared to the control RNA (total RNA). The RNA sequences that were within the selection criteria from the three biological repeats are clustered as heatmap enrichment (Fig. 2). Two hundred and fifteen Kanamycin B binding RNA sequences were identified under the growth condition without Kanamycin B (Fig. 2a, Table S1). Under the conditions in which cells were grown in the presence of 1 μM Kanamycin B, 230 Kanamycin B binding RNA sequences were obtained (Fig. 2b, Table S2). There were 134 RNA sequences identified in both conditions (Table S3) and some Kanamycin B binding RNAs were unique to each one of the conditions (Fig. 2c). When 0 μM and 1 μM of Kanamycin B was added, 81 and 96 unique genes were identified that interacted with Kanamycin B (Fig. 2c). This was probably due a change in the the overall transcription profile in the presence of 1 μM of Kanamycin B [46]. The Kanamycin B binding RNAs included rRNA, tRNA, ncRNA and mRNA including non-translated region RNA (Fig. 2d). Examples of Kanamycin B binding RNAs that are enriched more than tenfold in the presence of 1 μM Kanamycin B are shown in Table 1.

**Table 1:** Genes enriched greater than 10 fold in 1μM Kanamycin B pull down assay

**Transcript** **Name**	**Transcript ID**	**Type**	**Location**	**Product**	**Fold Change**
rrfF	b3272	rRNA	cytosol	5S ribosomal RNA	61.87
ibsC	b4665	mRNA	inner membrane	toxic peptide IbsC	60.49
rrfB	b3971	rRNA	cytosol	5S ribosomal RNA	56.83
rrfE	b4010	rRNA	cytosol	5S ribosomal RNA	56.12
trpT	b3761	tRNA	cytosol	tRNA-Trp(CCA)	49.42
rrfG	b2588	rRNA	cytosol	5S ribosomal RNA	48.15
ryfD	b4609	ncRNA	no annotation	small regulatory RNA RyfD	45.91
rrfD	b3274	rRNA	cytosol	5S ribosomal RNA	45.71
rrfH	b0205	rRNA	cytosol	5S ribosomal RNA	45.14
rrfC	b3759	rRNA	cytosol	5S ribosomal RNA	38.82
ffs	b0455	ncRNA	cytosol	signal recognition particle 4.5S RNA	35.04
ibsD	b4664	mRNA	inner membrane	putative toxic peptide IbsD	24.27
ssrS	b2911	ncRNA	no annotation	6S RNA	21.89
rrfA	b3855	rRNA	no annotation	5S ribosomal RNA	18.27
glmY	b4441	ncRNA	no annotation	small regulatory RNA GlmY	17.82
dicF	b1574	ncRNA	no annotation	Qin prophage; small regulatory RNA DicF	16.18
alaW	b2397	tRNA	cytosol	tRNA-Ala(GGC)	15.94
sibD	b4447	ncRNA	no annotation	small RNA SibD	14.08
fliG	b1939	mRNA	inner membrane, cell projection	flagellar motor switch protein FliG	12.73
ryjA	b4459	ncRNA	no annotation	small RNA RyjA	11.73
insA2	b0265	mRNA	cytosol	IS1 protein InsA	11.64
fnrS	b4699	ncRNA	no annotation	small regulatory RNA FnrS	11.32
ves	b1742	mRNA	cytosol	HutD family protein Ves	11.09
valV	b1665	tRNA	cytosol	tRNA-Val(GAC)	10.53
cydH	b4602	mRNA	inner membrane	cytochromebd-I ubiquinol oxidase accessory subunit CydH	10.05

### Functional classification of Kanamycin B binding RNA

**Fig. 3:**
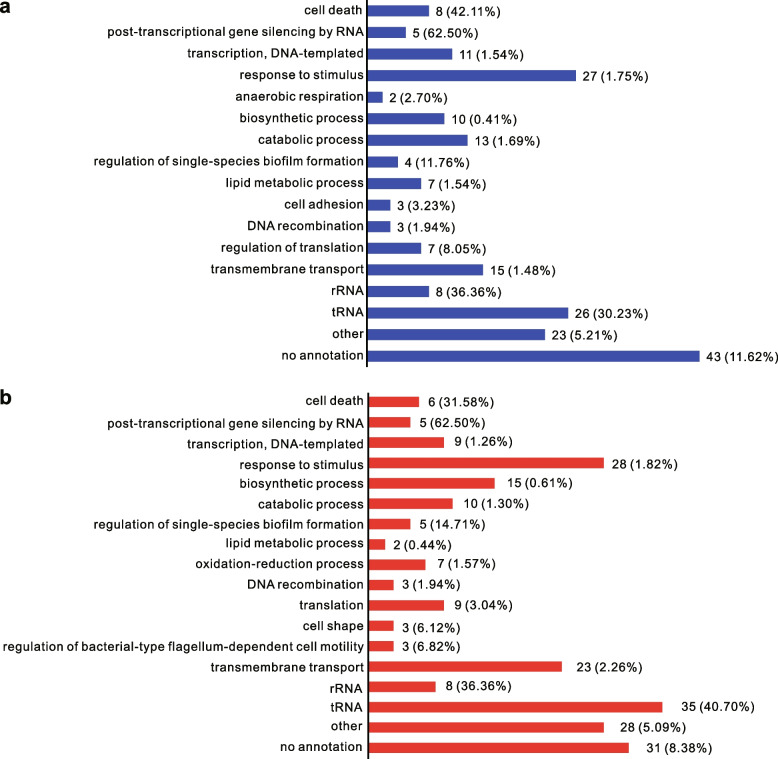
Functional classification of enriched genes in Kanamycin B pull down assay. ECOCYC database was used for the functional classification of the genes. **a** Functional classification of 215 enriched genes in 0 μM Kanamycin B pull down assay. **b** Functional classification of 230 enriched genes in 1 μM Kanamycin B pull down assay. The percentage of the number of each enriched gene group in the whole functional classification gene number in *E. coli* is shown in brackets

To investigate the function of the Kanamycin B binding RNA associated genes, DAVID [47] function analysis was used, based on ECOCYC database [48, 49]. Figure 3a and Table S1 shows the function of the Kanamycin B binding RNA associated genes classified into known or predicted functional groups or unannotated groups under the culture condition in the absence of Kanamycin B. These genes encode proteins that are known or predicted to be involved in the following cellular functions: programmed cell death, post-transcriptional gene silencing by RNA transcription, response to stimulus, anaerobic respiration, biosynthetic process, catabolic process, regulation of single-species biofilm formation, lipid metabolic process, cell adhesion, DNA recombination, regulation of translation, transmembrane transport, rRNA, tRNA and others with no annotation. In cultures with 1 μM Kanamycin B, the function of the Kanamycin B binding RNA associated genes fall into the same classification as Fig. 3a with one additional group of 8 genes that encode proteins involved in oxidation and reduction processes (Fig. 3b), suggesting that Kanamycin B maybe associated with or involved with the cellular oxidation and reduction process. In the GO term functional annotation in the ECOCYC database, the number of genes that belong to each functional group in *E. coli* is known [48]. The percentage of the numbers of the enriched genes (pull down RNA) associated with each functional group of the total number of genes involved in each gene function in *E. coli* was calculated. This analysis shows that the functions of the pull down RNA largely fall into cell death (42.11% and 31.58% for 0 and 1 μM Kanamycin B respectively) and post-transcriptional gene silencing by RNA groups (62.5% for 0 and 1 μM Kanamycin B) (Fig. 3a and b), suggesting that Kanamycin B cellular function is related to cell death and post-transcriptional gene silencing by RNA.

### Measurement of affinity binding of the identified Kanamycin B pull down RNAs with Kanamycin B

**Fig. 4:**
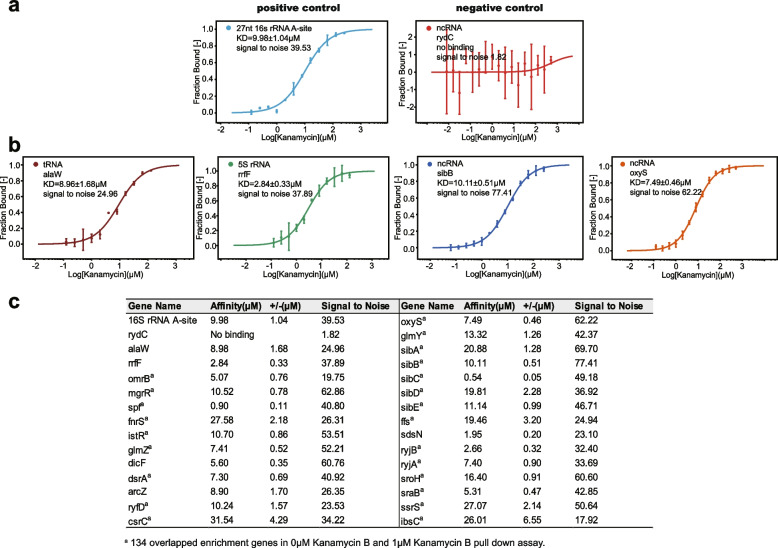
Kanamycin B binding measurements to RNA using MST. **a** Binding curve generated by MST for Kanamycin B binding to the positive and negative control RNAs. Error bars were calculated from three independent biological repeats with duplicate measurements of each sample. A signal-to-noise ratio of more than 5 is desirable, while more than 12 reflects an excellent assay. **b** Binding curve generated by MST for binding of Kanamycin B to tRNA, 5sRNA or ncRNA. **c** Binding affinity of 28 enriched RNAs with Kanamycin B measured by MST

The Kanamycin B binding RNAs identified by using Kanamycin B Sepharose beads included tRNA, rRNA, ncRNA and mRNA (Fig. 2d and Table S1 and S2). The mRNAs identified contained sequences of various lengths, the majority of which are over 1000 nt but a few are shorter, such as ibsC mRNA and smaller ncRNA, rRNA and tRNAs were also identified of ~ 200 nt, that are feasible to make in vitro. To verify that the identified RNAs bind to Kanamycin B in vitro by an alternative method, MicroScale Thermophoresis (MST) was then used to measure binding of Kanamycin B to 24 ncRNA, 2 tRNA, 1 rRNA, 1 ibsC mRNA. The RNAs were prepared and labeled with fluorescein-5-thiosemicarbazide [50]. The binding measurements were made on a Monolith NT.115 system (NanoTemper Technologies) [51, 52]. On titration of Kanamycin B increased binding was observed for 24 ncRNA, suggesting the formation of a Kanamycin B-RNA complex. The dissociation constants (KD) for Kanamycin B-RNA complex are shown in Fig. 4. Binding to the 16S rRNA (A site) was observed with a KD of 9.98 μM. Since rRNA ribosome A site is a known Kanamycin B binding site, it serves as a positive control for the binding measurement (Fig. 4a) [2]. The ncRNA rydC was not enriched in the pull-down experiment, and no binding with Kanamycin B was observed. This RNA can therefore be considered as a negative control for the binding measurement (Fig. 4a). 5S rRNA is among the highly enriched in the pull-down experiments and binds to Kanamycin B with KD of 2.84 μM. Two tRNAs also bind Kanamycin B (Fig. 4b). The binding affinities of the tested RNAs for Kanamycin B is tabulated in Fig. 4c (Fig. S1). Thus, these results show a good correlation between the results of the pull-down assay and the in vitro MST binding data.

### Chemical probing of ibsC mRNA in the presence of Kanamycin B

**Fig. 5:**
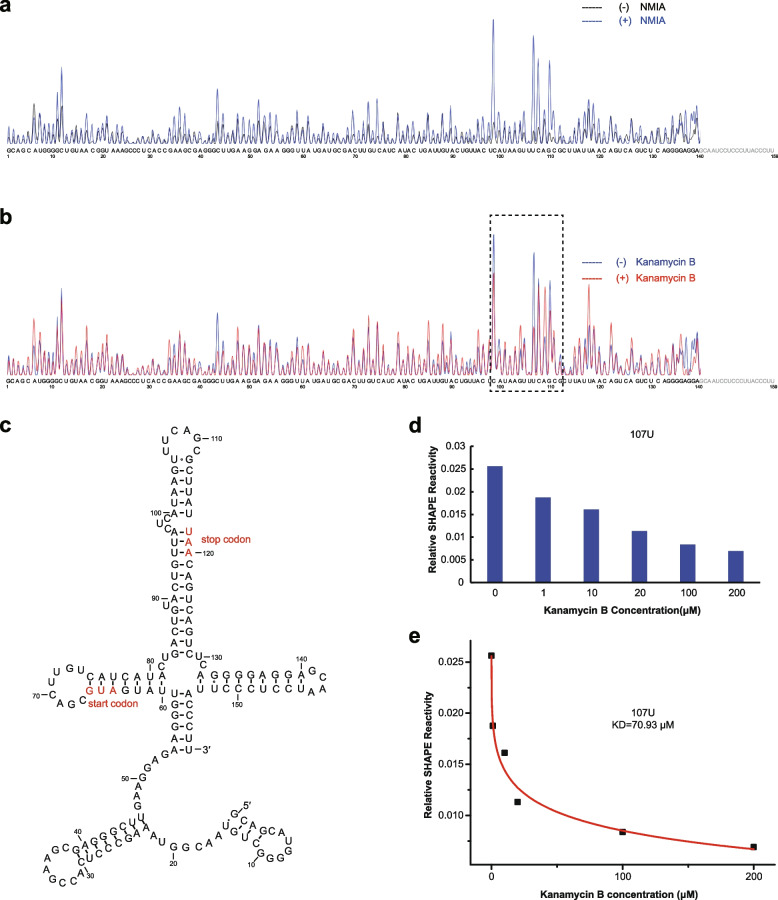
SHAPE probing of ibsC mRNA on Kanamycin B titration. **a** Electropherogram of NMIA-modified RNA with NMIA ([ +] NMIA, blue trace), compared with that of unmodified RNA ([ −] NMIA, black trace). **b** Analysis of NMIA modification electropherogram of ibsC RNA with 20 μM Kanamycin B (red trace), compared with 0 μM Kanamycin B (blue trace). **c** The secondary structure of the ibsC RNA [53]. **d** Histogram of NMIA modification of the in vitro transcribed RNA nucleotides (58–62) on titration of Kanamycin B. The reactivity of the nucleotide is calculated as the ratio of the height of the nucleotide to the height of all nucleotides. **e** Kanamycin B binding measured through changes in NMIA reactivity at the 107U position in the RNA, on titration with Kanamycin B (data taken from Figure S2)

The ibsC mRNA that encodes a peptide toxin, shows 61 fold of enrichment in the pull down assay (Table 1) with a binding affinity of 26 μM to Kanamycin B by MST measurement (Fig. 4c). The secondary structure of the ibsC mRNA has been reported [53]. To further investigate the effect of Kanamycin B binding on the structure of ibsC RNA, we performed selective 2′-hydroxyl acylation analyzed by primer extension (SHAPE) on ibsC mRNA on titration of Kanamycin B by capillary electrophoresis with fluorescence detection. In the absence of Kanamycin B, our data shows that the ibsC RNA folds into a secondary structure that is largely similar to the reported structure from enzymatic and chemical footprinting (Fig. 5a) [53]. In response to Kanamycin B titration, the reactivity of some nucleotides show progressive changes (Fig. 5b and Fig. S2). In particular, a clear reduction in reactivity is observed on Kanamycin B titration at nucleotides in the region of 107U within a loop located before the stop codon of the peptide (Fig. 5c and d). The changes in reactivity at certain nucleotides may be due to Kanamycin B binding to the RNA at these nucleotides or through a Kanamycin B induced a structural transition in the RNA, both of which would be indistinguishable. Kanamycin B binding sites or structural changes upon binding occurring before the stop codon of ibsC mRNA indicate that Kanamycin B binding may affect translation of the IbsC peptide toxin. The reduction in reactivity as measured at 107U was consistent with a KD of Kanamycin B for the RNA of ~ 70 μM (Fig. 5e), while MST measurement show binding affinity of 26 μM (Fig. 4c).

## Discussion

Kanamycin B, as an aminoglycoside antibiotic is known to inhibit translation by binding to the A site of the ribosome. Here we have identified over two hundred Kanamycin B binding RNA sequences genome-wide in *E. coli* in this study (Table 1, Table S1 and S2). Kanamycin B immobilized Sepharose beads were used to pull-down RNA that was subsequently subjected to RNA-seq. Binding of these small RNAs was further verified by MST (Fig. 4). Functional classification analysis of the Kanamycin B binding RNA sequences revealed that Kanamycin B binding RNA may participate in a wide arrange of biological processes (Fig. 3), suggesting Kanamycin B may have additional non-antibiotic functions through binding to *E. coli* RNAs.

The Kanamycin B binding RNAs were identified by the enrichment of the RNA in pull-down experiment compared to the total RNA control. However, the well-known Kanamycin B binding site 16S RNA the A-site in the ribosome was not identified by the enrichment criteria. This may have been due to disruption of the ribosomal A-site during the RNA purification step of the pull-down assay. There were 35 tRNAs identified as Kanamycin B binding RNAs in this study of which two tRNA transcripts have been verified to bind to Kanamycin B by MST. Aminoglycoside binding to tRNAs in vitro have been previously reported [54]. Together with the observations in this study, Kanamycin B binding to tRNA may have a biological function that still requires further investigation.

In order to investigate if there is a commonly shared Kanamycin B binding motif among all the identified pull down RNA, we used CMfinder (a covariance model based RNA motif finding algorithm) to predict RNA motif. No main common covariance motif was found although we noticed shared stem loops with different specific sequences. In the study of in vitro selection of unnatural RNA against Kanamycin B [45], they also found a stem loop structural motif but the sequences of the RNA were totally different from the known sequences of the Kanamycin B binding site or the A-site in the ribosome. They suggested that the Kanamycin B-binding region has a shape-specific stem loop with a specific sequence. These studies collectively suggest that Kanamycin B binding may favour a stem loop in RNA but with different sequences (Fig. S3). The data from our SHAPE analysis of ibsC RNA on titration of Kanamycin B suggest that the Kanamycin B binding site may be located within the stem loop (Fig. 5). Kanamycin B binding to the ibsC mRNA specifically at the stem loop before the stop codon may effect translation of the peptide toxin or ibsC and sibC binding and consequently effect their role in cell death [53].

Bacterial biofilms are complex multicellular aggregates that form in response to environmental challenges. There have been reports that some aminoglycoside antibiotics induce bacterial biofilm formation [24]. Thirty-three small ncRNAs involved in biofilm formation, motility, and fimbriae formation in *E. coli* have been identified [55]. Ten of the thirty-three small ncRNAs were also identified in this study and their in vitro binding to Kanamycin B was measured by MST. Since Kanamycin B is a secondary metabolite natural product that is synthesised to protect the bacteria within its local environment, it may also function as a small regulator molecule. These observations together point to the possibility that Kanamycin B may serve as a signaling molecule in the complex process of antibiotic induced bacterial biofilm formation.

Small metabolite molecules have been reported to regulate gene expression by binding to untranslated RNA [56, 57]. Aminoglycosides sensing riboswitches regulate the translation of the aminoglycoside resistance genes [12, 58]. Aminoglycosides have also been shown to bind to the nucleolytic ribozymes twister ribozyme and change their activity [19–21, 23, 59]. These individual evidences suggested that aminoglycosides have non-antibiotic cellular functions. However, here we have reported genome-wide identification of Kanamycin B binding RNAs that are unknown before. The results presented in this study pave foundation for further understanding of novel aminoglycoside cellar functions.

## Materials and methods

### Bacterial growth and total RNA extraction

Total RNA was extracted from *E.coli* K-12 derived JM109 strain grown in LB to log phase with the Trizol method (Invitrogen, USA). DNase I (TakaRa, Japan) was used for RNA purification. The RNA quality was assessed by formaldehyde agarose gel electrophoresis and was quantitated spectrophotometrically (NanoDrop 2000).

### Immobilization of kanamycin B to Sepharose beads

Kanamycin B was immobilized on Pierce™ NHS-Activated Agarose (Thermo Scientific™). For this, 0.05 g dry agarose was swollen in MilliQ-H_2_O and twice washed with coupling buffer (0.1 M sodium phosphate, 0.15 M NaCl, pH7.2). After a second wash with coupling buffer, the agarose was mixed 1:2 with 10 mM Kanamycin B solution in coupling buffer. The reaction was protected from light and incubated overnight at 4 °C on an H5600 rotator (Labnet). Afterwards, unreacted Kanamycin B was washed, away with ~ 2 volumes of coupling buffer. Finally, quenching buffer was added (1 M Tris, pH7.5/8.0), and incubated for 20 min. A non-derivatized column (mock), consisting of NHS-Activated Agarose that had been treated with coupling buffer without Kanamycin B.

### Kanamycin B pull-down Assay

RNA was folded by heating the mixture to 95 °C for 5 min and placed on ice water for 5 min, the volume was adjusted to 1 column volume (CV, 500 μl) with 1 × binding buffer (40 mM HEPES pH 7.4, 100 mM KCl, 2.5 mM MgCl_2_, 0.1% NP40), respectively. For depletion of RNAs able to bind the affinity matrix, the RNA was first incubated for 30 min with 1 CV of the mock column. After negative selection, unbound RNAs were added to 1 CV Kanamycin B-coupled agarose and incubated for 30 min at room temperature. Next, the column was washed with 20 CV binding buffer and specific bound RNAs were eluted by 5 mM Kanamycin B in 1 × binding buffer. The RNA precipitated with an equal volume of isopropanol, 1/300 volume of Glycoblue, 0.1 volume of 3 M NaOAc pH 5.2. RNA pellet was washed in 1 ml 70% EtOH, and pellets were resuspended in 10 μl H_2_O.

### Library preparation

The cDNA libraries are constructed using the NEBNext Ultra II Directional RNA Library Prep Kit (New England Biolabs). The library preparations were constructed according to the manufacturer′ s protocol. Three biological replicates of each library were sequenced on an Illumina HiSeq X Ten (Illumina, San Diego, CA, USA) by AZENTA (Suzhou, China). RNA-seq data were uploaded to the Sequence Read Archive of the National Center for Biotechnology Information (accession number: PRJNA756617, PRJNA760293).

### Sequence reads mapping and assembly

Firstly, raw reads in the fastq format were filtered to remove reads containing adaptors, reads containing poly-N and low quality reads using Trim Galore [60]. At the same time, Q20, Q30, GC-content and sequence duplication levels in the clean data were calculated. All of the subsequent analyses were carried out using high quality clean reads. The clean reads were then mapped to the *E. coli* str. K-12 substr. MG1655, genome assembly (NCBI: NC_000913.3) by using bowtie2 v2.4.2 [61].

The DEseq2(1.28.0) [62] package was applied to filter the RNAs with fold change over inputs ≥ 2 and *P* value < 0.05 were defined as enriched.

### Identification of Kanamycin B binding RNA

EcoCyc is a literature-based database for *E.coli*, containing information about its genome, gene regulation, and metabolic pathways. All enriched genes were assigned a functional category based on available databases (EcoCYC [48, 49], DAVID [47]).

### Principal component analysis

PCA was analysed with the plotPCA function in DESEQ2 package. The scores of the first and second principal components were plotted into two-dimensional space to represent the spatial relationships within the repeat samples for visualization [63].

### Micro Scale Thermophoresis (MST)

The RNAs were produced by in vitro transcription using the appropriate DNA templates and T7 RNA Polymerase (T7 RNA Polymerase was produced in our laboratory). The corresponding DNA templates were prepared by PCR amplifying the genomic DNA of *E. coli* JM109 using specific primers with the promoter sequence (TAATACGACTCACTATAGG) for T7 RNA polymerase at the 5′-end. PCR amplified using Phanta Max Super-Fidelity DNA Polymerase (Vazyme) according to the supplier’s instructions. After ethanol precipitation, the DNA template was used for in vitro transcription. In vitro transcription was carried out in 40 mM Tris- HCl (pH 7.9), 20 mM MgCl_2_, 0.01%(v/v) TritonX-100 and 2 mM sperdine at 37 °C for 4 h. The RNAs was were purified by denaturing polyacrylamide gel electrophoresis (PAGE), and isolated from the gel by crush-soaking in 300 mM NaOAC and 1 mM EDTA. After precipitation with ethanol, the molarity of RNAs was determined by spectrophotometric measurement using NanoDrop 1000 Spectrophotometer (Thermo Scientific). Purified RNA was labeled with fluorescein- 5-thiosemicarbazide as previously described [50]. The Fluorescein-RNA was annealed by heating to 95 °C for 5 min and then cooled to room temperature. The prepared RNA and antibiotics were both incubated in 1 × binding buffer (40 mM HEPES pH 7.4, 100 mM KCl, 2.5 mM MgCl_2_) at room temperature. MST experiments were conducted in triplicate on a Monolith NT.115 system (NanoTemper Technologies) [51, 52].

### SHAPE

The ibsC RNA with 3′ linkers transcribed by T7 RNA polymerase was subjected to SHAPE analysis on titration of Kanamycin B. A series of concentrations of Kanamycin B were used with approximately 20 pmol of RNA in 1 × binding buffer (40 mM HEPES pH 7.4, 100 mM KCl, 2.5 mM MgCl_2_), and RNA was probed by 2 μl of freshly prepared 65 mM N-methylisatoic anhydride (NMIA, TCI) in DMSO at 25 °C for 2.5 h [11]. Control RNA samples were prepared with DMSO without NMIA. After ethanol precipitation, the RNA was used for cDNA synthesis by SuperScript IV Reverse Transcription Kit (Invitrogen) with 2 pmol FAM labeled primer. DNA sequences were analyzed by ABI 3730 DNA sequencer. Sequence markers were produced by reverse transcription of the RNA using a SuperScriptTM IV Reverse Transcription Kit with ddNTPs. The reactivity of each nucleotide is calculated as the ratio of the height of the each nucleotide to the height of all nucleotides and normalized against the control RNA sample with no added NMIA.

## Supplementary Information

**Additional file 1: Table S1.** Fold change and gene functions of 215 enrichment genes in 0 μM Kanamycin B pull down assay.

**Additional file 2: Table S2.** Fold change and gene functions of 230 enrichment genes in 1 μM Kanamycin B pull down assay.

**Additional file 3: Table S3.** 134 overlapped enrichment genes in 0 μM Kanamycin B and 1 μM Kanamycin B pull down assay.

**Additional file 4: Fig. S1.** Binding curve generated by MST for binding of Kanamycin B to 24 enriched RNA with Kanamycin B measured by MST.

**Additional file 5: Fig. S2.** Electropherogram of NMIA-modification on ibsC RNA on Kanamycin B titration.

**Additional file 6: Fig. S3.** Comparison of a secondary structure of RNA against Kanamycin B. a stem loop of ibsC. b stem loop of spf. c stem loop of dsrA. d stem loop of the 16 s rRNA A-site model.

## Data Availability

The RNA-Seq data supporting the results of this article have been uploaded to the Sequence Read Archive of NCBI (National Center for Biotechnology Information). It could be accessed via the NCBI SRA database with accession number PRJNA756617, PRJNA760293.

## Abbreviations

*E.coli*: *Escherichia coli*
SELEX: Systematic evolution of ligands by exponential enrichment
PCA: Principal component analysis
DEGs: Differentially expressed genes
MST: MicroScale thermophoresis
KD: Dissociation constants
SHAPE: Selective 2′ -hydroxyl acylation analyzed by primer extension
NMIA: N-methylisatoic anhydride

## References

[1] Davies J, Davis B (1968). Misreading of ribonucleic acid code words induced by aminoglycoside antibiotics. The effect of drug concentration. J Biol Chem.

[2] Fourmy D, Recht M, Blanchard S, Puglisi J (1996). Structure of the A site of Escherichia coli 16S ribosomal RNA complexed with an aminoglycoside antibiotic. Science (New York, NY).

[3] Carter AP, Clemons WM, Brodersen DE, Morgan-Warren RJ, Ramakrishnan V (2000). Functional insights from the structure of the 30S ribosomal subunit and its interactions with antibiotics. Nature.

[4] Saswati S, Chattopadhyay MK, Hans-Peter G (2013). The multifaceted roles of antibiotics and antibiotic resistance in nature. Front Microbiol.

[5] Davies J, Davies D (2010). Origins and Evolution of Antibiotic Resistance. Microbiol Mol Biol Rev.

[6] Schroeder R, Waldsich C, Wank H (2000). Modulation of RNA function by aminoglycoside antibiotics. EMBO J.

[7] Flatt PM, Mahmud T (2007). Biosynthesis of aminocyclitol-aminoglycoside antibiotics and related compounds. Nat Prod Rep.

[8] Daikos GL, Lolans VT, Jackson G (1991). First-exposure adaptive resistance to aminoglycoside antibiotics in vivo with meaning for optimal clinical use. Antimicrob Agents Chemother.

[9] Xiong Y-Q, Caillon J, Kergueris MF, Drugeon H, Baron D, Potel G, Bayer AS (1997). Adaptive resistance of Pseudomonas aeruginosa induced by aminoglycosides and killing kinetics in a rabbit endocarditis model. Antimicrob Agents Chemother.

[10] Wistrand-Yuen E, Knopp M, Hjort K, Koskiniemi S, Berg OG, Andersson DI (2018). Evolution of high-level resistance during low-level antibiotic exposure. Nat Commun.

[11] Jia X, Zhang J, Sun W, He W, Jiang H, Chen D, Murchie A (2013). Riboswitch control of aminoglycoside antibiotic resistance. Cell.

[12] Wang S, He W, Sun W, Zhang J, Chang Y, Chen D, Murchie A (2019). Integron-Derived Aminoglycoside-Sensing Riboswitches Control Aminoglycoside Acetyltransferase Resistance Gene Expression. Antimicrob Agents Chemother.

[13] Chen D, Murchie A (2014). An aminoglycoside sensing riboswitch controls the expression of aminoglycoside resistance acetyltransferase and adenyltransferases. Biochem Biophys Acta.

[14] He W, Zhang X, Zhang J, Jia X, Zhang J, Sun W, Jiang H, Chen D, Murchie A (2013). Riboswitch control of induction of aminoglycoside resistance acetyl and adenyl-transferases. RNA Biol.

[15] Borovinskaya MA, Pai RD, Zhang W, Schuwirth BS, Holton JM, Hirokawa G, Kaji H, Kaji A, Cate JHD (2007). Structural basis for aminoglycoside inhibition of bacterial ribosome recycling. Nat Struct Mol Biol.

[16] Mei HY, Mack DP, Galan AA, Halim NS, Heldsinger A, Loo JA, Moreland DW, Sannes-Lowery KA, Sharmeen L, Truong HN (1997). Discovery of selective, small-molecule inhibitors of RNA complexes—1. The tat protein/TAR RNA complexes required for HIV-1 transcription. Bioorg Med Chem.

[17] Zapp ML, Stern S, Green MR (1993). Small molecules that selectively block RNA binding of HIV-1 Rev protein inhibit Rev function and viral production. Cell.

[18] von Ahsen U, Davies J, Schroeder R (1991). Antibiotic inhibition of group I ribozyme function. Nature.

[19] Stage TK, Hertel KJ, Uhlenbeck OC (1995). Inhibition of the hammerhead ribozyme by neomycin. RNA.

[20] Earnshaw DJ, Gait MJ (1998). Hairpin ribozyme cleavage catalyzed by aminoglycoside antibiotics and the polyamine spermine in the absence of metal ions. Nucleic Acids Res.

[21] Zhang J, Liu G, Sun W, Chen D, Murchie AI (2021). Aminoglycoside antibiotics can inhibit or activate twister ribozyme cleavage. FEBS J.

[22] Rogers J, Chang AH, von Ahsen U, Schroeder R, Davies J (1996). Inhibition of the self-cleavage reaction of the human hepatitis delta virus ribozyme by antibiotics. J Mol Biol.

[23] Wrzesinski J, Błaszczyk L, Wrońska M, Kasprowicz A, Stokowa-Sołtys K, Nagaj J, Szafraniec M, Kulinski T, Jeżowska-Bojczuk M, Ciesiołka J (2013). Mapping the interactions of selected antibiotics and their C u2+ complexes with the antigenomic delta ribozyme. FEBS J.

[24] Hoffman L, D'Argenio D, Maccoss M, Zhang Z, Jones R, Miller S (2005). Aminoglycoside antibiotics induce bacterial biofilm formation. Nature.

[25] Ysern P, Clerch B, Castańo M, Gibert I, Barbé J, Llagostera M (1990). Induction of SOS genes in Escherichia coli and mutagenesis in Salmonella typhimurium by fluoroquinolones. Mutagenesis.

[26] Miller C, Thomsen LE, Gaggero C, Mosseri R, Ingmer H, Cohen SN (2004). SOS response induction by ß-lactams and bacterial defense against antibiotic lethality. Science.

[27] Shaw KJ, Miller N, Liu X, Lerner D, Wan J, Bittner A, Morrow BJ (2003). Comparison of the changes in global gene expression of Escherichia coli induced by four bactericidal agents. Microbial Physiology.

[28] Cohen SE, Walker GC (2010). The transcription elongation factor NusA is required for stress-induced mutagenesis in Escherichia coli. Curr Biol.

[29] Baharoglu Z, Mazel D (2011). Vibrio cholerae triggers SOS and mutagenesis in response to a wide range of antibiotics: a route towards multiresistance. Antimicrob Agents Chemother.

[30] Guerin É, Cambray G, Sanchez-Alberola N, Campoy S, Erill I, Da Re S, Gonzalez-Zorn B, Barbé J, Ploy M-C, Mazel D (2009). The SOS response controls integron recombination. Science.

[31] Ellington AD, Szostak JW (1992). Selection in vitro of single-stranded DNA molecules that fold into specific ligand-binding structures. Nature.

[32] Joyce GF (1989). Amplification, mutation and selection of catalytic RNA. Gene.

[33] Joyce G, Orgel L (1988). Non-enzymatic template-directed synthesis on RNA random copolymers: Poly (C, A) templates. J Mol Biol.

[34] Schmidt FJ (1999). Ribozymes–why so many, why so few?. Mol Cells.

[35] Eaton BE, Gold L, Hicke BJ, Janjié N, Jucker FM, Sebesta DP, Tarasow TM, Willis MC, Zichi DA (1997). Post-SELEX combinatorial optimization of aptamers. Bioorg Med Chem.

[36] Hermann T, Patel DJ (2000). Adaptive recognition by nucleic acid aptamers. Science.

[37] Lato SM, Boles AR, Ellington AD (1995). In vitro selection of RNA lectins: using combinatorial chemistry to interpret ribozyme evolution. Chem Biol.

[38] Lorsch JR, Szostak JW (1996). Chance and necessity in the selection of nucleic acid catalysts. Acc Chem Res.

[39] Suess B, Hanson S, Berens C, Fink B, Schroeder R, Hillen W (2003). Conditional gene expression by controlling translation with tetracycline-binding aptamers. Nucleic Acids Res.

[40] Groher F, Bofill-Bosch C, Schneider C, Braun J, Jager S, Geißler K, Hamacher K, Suess B (2018). Riboswitching with ciprofloxacin—development and characterization of a novel RNA regulator. Nucleic Acids Res.

[41] Lato SM, Ellington AD (1996). Screening chemical libraries for nucleic-acid-binding drugs by in vitro selection: a test case with lividomycin. Mol Diversity.

[42] Wallis MG, von Ahsen U, Schroeder R, Famulok M (1995). A novel RNA motif for neomycin recognition. Chem Biol.

[43] Wang Y, Rando RR (1995). Specific binding of aminoglycoside antibiotics to RNA. Chem Biol.

[44] Jiang L, Patel DJ (1998). Solution structure of the tobramycin–RNA aptamer complex. Nat Struct Biol.

[45] Kwon M, Chun S, Jeong S, Yu J (2001). In vitro selection of RNA against kanamycin B. Mol Cells.

[46] Chang Y, Zhang X, Murchie AIH, Chen D (2022). Transcriptome profiling in response to Kanamycin B reveals its wider non-antibiotic cellular function in Escherichia coli. Front Microbiol.

[47] Huang DW, Sherman BT, Lempicki RA (2009). Systematic and integrative analysis of large gene lists using DAVID bioinformatics resources. Nat Protoc.

[48] Keseler IM, Mackie A, Santos-Zavaleta A, Billington R, Bonavides-Martínez C, Caspi R, Fulcher C, Gama-Castro S, Kothari A, Krummenacker M (2017). The EcoCyc database: reflecting new knowledge about Escherichia coli K-12. Nucleic Acids Res.

[49] Karp PD, Billington R, Caspi R, Fulcher CA, Latendresse M, Kothari A, Keseler IM, Krummenacker M, Midford PE, Ong Q (2019). The BioCyc collection of microbial genomes and metabolic pathways. Brief Bioinform.

[50] Wu T, Ruan K, Liu W (1996). A fluorescence-labeling method for sequencing small RNA on polyacrylamide gel. Nucleic Acids Res.

[51] Entzian C, Schubert T (2016). Studying small molecule–aptamer interactions using MicroScale Thermophoresis (MST). Methods.

[52] Moon M, Hilimire T, Sanders A, Schneekloth J (2018). Measuring RNA-Ligand Interactions with Microscale Thermophoresis. Biochemistry.

[53] Han K, Kim K, Bak G, Park H, Lee Y (2010). Recognition and discrimination of target mRNAs by Sib RNAs, a cis-encoded sRNA family. Nucleic Acids Res.

[54] Walter F, Pütz J, Giegé R, Westhof E (2002). Binding of tobramycin leads to conformational changes in yeast tRNAAsp and inhibition of aminoacylation. EMBO J.

[55] Bak G, Lee J, Suk S, Kim D, Young Lee J, Kim KS, Choi BS, Lee Y (2015). Identification of novel sRNAs involved in biofilm formation, motility and fimbriae formation in Escherichia coli. Sci Rep.

[56] Sun W, Zhang X, Chen D, Murchie A (2020). spe2Interactions between the 5' UTR mRNA of the gene and spermidine regulate translation in. RNA (New York, NY).

[57] Zhang X, Sun W, Chen D, Murchie A (2020). sam1Interactions between SAM and the 5' UTR mRNA of the gene regulate translation in. RNA (New York, NY).

[58] Zhang J, Liu G, Zhang X, Chang Y, Wang S, He W, Sun W, Chen D, Murchie A (2020). Aminoglycoside riboswitch control of the expression of integron associated aminoglycoside resistance adenyltransferases. Virulence.

[59] Lovett PS, Rogers EJ (1996). Ribosome regulation by the nascent peptide. Microbiol Rev.

[60] Utturkar S, Dassanayake A, Nagaraju S, Brown SD. Bacterial Differential Expression Analysis Methods. In: Himmel M, Bomble Y. (eds) Metabolic Pathway Engineering. Methods in Molecular Biology, vol 2096. New York: Humana; 2020. 10.1007/978-1-0716-0195-2_8. 10.1007/978-1-0716-0195-2_8 32720149

[61] Langdon WB (2015). Performance of genetic programming optimised Bowtie2 on genome comparison and analytic testing (GCAT) benchmarks. BioData mining.

[62] Love MI, Huber W, Anders S (2014). Moderated estimation of fold change and dispersion for RNA-seq data with DESeq2. Genome Biol.

[63] Long J, Zhang J, Zhang X, Wu J, Du C (2020). Genetic Diversity of Common Bean (Phaseolus vulgaris L.) Germplasm Resources in Chongqing, Evidenced by Morphological Characterization. Front Genet.

