# Supplementary material for: Genome-wide identification of Kanamycin B binding RNA in Escherichia coli
Source: BMC Genomics. 2023 Mar 16;24:120. doi: 10.1186/s12864-023-09234-3 (PMC10018874; doi:10.1186/s12864-023-09234-3)
Supplement: Supplementary file 1 — Additional file 1: Table S1. Fold change and gene functions of 215 enrichment genes in 0 μM Kanamycin B pull down assay. [file 12864_2023_9234_MOESM1_ESM.docx]

**Table S1. Fold change and gene functions of 215 enrichment genes in 0μM Kanamycin B pull down assay.**

^a^ 134 overlapped enrichment genes in 0μM Kanamycin B and 1μM Kanamycin B pull down assay.

| **Transcript Name** | **Transcript ID** | **Type** | **Location** | **Product** | **Fold Change** |
| --- | --- | --- | --- | --- | --- |
| **cell death** | | |  |  |  |
| ibsC^a^ | b4665 | mRNA | inner membrane | toxic peptide IbsC | 44.06 |
| ibsD^a^ | b4664 | mRNA | inner membrane | putative toxic peptide IbsD | 38.80 |
| ibsB | b4668 | mRNA | inner membrane | putative toxic peptide IbsB | 16.88 |
| ibsA | b4667 | mRNA | inner membrane | toxic peptide IbsA | 16.31 |
| ldrC^a^ | b4423 | mRNA | inner membrane | small toxic polypeptide LdrC | 3.95 |
| ldrA^a^ | b4419 | mRNA | inner membrane | small toxic polypeptide LdrA | 3.64 |
| shoB^a^ | b4687 | mRNA | inner membrane | toxic peptide ShoB | 3.35 |
| ldrB | b4421 | mRNA | inner membrane | small toxic polypeptide LdrB | 2.18 |
| **post-transcriptional gene silencing by RNA** | | | | |  |
| sibD^a^ | b4447 | ncRNA | no annotation | small RNA SibD | 9.92 |
| sibC^a^ | b4446 | ncRNA | no annotation | small regulatory RNA antitoxin SibC | 4.70 |
| sibE^a^ | b4611 | ncRNA | no annotation | small RNA SibE | 3.64 |
| sibB^a^ | b4437 | ncRNA | no annotation | small RNA SibB | 3.44 |
| sibA^a^ | b4436 | ncRNA | no annotation | small RNA SibA | 3.27 |
| **transcription, DNA-templated** | | |  |  |  |
| lrhA^a^ | b2289 | mRNA | cytosol | DNA-binding transcriptional dual regulator LrhA | 7.25 |
| gutM | b2706 | mRNA | inner membrane | DNA-binding transcriptional activator GutM | 7.16 |
| frvR | b3897 | mRNA | inner membrane | putative transcriptional regulator FrvR | 3.40 |
| pyrL^a^ | b4246 | mRNA | cytosol | pyrBIoperon leader peptide | 3.35 |
| cecR^a^ | b0796 | mRNA | cytosol | DNA-binding transcriptional dual regulator CecR | 2.49 |
| malI | b1620 | mRNA | cytosol | DNA-binding transcriptional repressor MalI | 2.32 |
| hupB^a^ | b0440 | mRNA | cytosol | DNA-binding protein HU-β | 2.29 |
| fucR | b2805 | mRNA | cytosol | DNA-binding transcriptional activator FucR | 2.24 |
| yfiE | b2577 | mRNA | cytosol | putative LysR-type DNA-binding transcriptional regulator YfiE | 2.18 |
| sgcR | b4300 | mRNA | cytosol | KpLE2 phage-like element; putative DNA-binding transcriptional regulator SgcR | 2.17 |
| thrL^a^ | b0001 | mRNA | cytosol | throperon leader peptide | 2.09 |
| **response to stimulus** | |  |  |  |  |
| glmY^a^ | b4441 | ncRNA | no annotation | small regulatory RNA GlmY | 14.62 |
| yjjQ | b4365 | mRNA | cytosol | DNA-binding transcriptional repressor YjjQ | 12.68 |
| ynhF^a^ | b4602 | mRNA | inner membrane | cytochromebd-I ubiquinol oxidase accessory subunit CydH | 10.12 |
| ftsI^a^ | b0084 | mRNA | inner membrane | peptidoglycan DD-transpeptidase FtsI | 5.69 |
| yqaE^a^ | b2666 | mRNA | inner membrane | Pmp3 family protein YqaE | 4.41 |
| dinQ^a^ | b4613 | mRNA | inner membrane | membrane toxin DinQ | 4.02 |
| oxyS^a^ | b4458 | ncRNA | bacterial nucleoid, cytosol | small regulatory RNA OxyS | 3.74 |
| symE | b4347 | mRNA | cytosol | toxic protein SymE | 3.70 |
| yphB^a^ | b2544 | mRNA | cytosol | putative aldose 1-epimerase YphB | 3.57 |
| mgtS^a^ | b4599 | mRNA | inner membrane | small protein MgtS | 3.25 |
| ecnB^a^ | b4411 | mRNA | inner membrane, outer membrane | bacteriolytic entericidin B lipoprotein | 3.20 |
| mdtP | b4080 | mRNA | outer membrane | putative multidrug efflux pump outer membrane channel | 3.11 |
| rmf^a^ | b0953 | mRNA | cytosol | ribosome modulation factor | 2.92 |
| acrZ^a^ | b0762 | mRNA | cytosol, outer membrane, inner membrane | multidrug efflux pump accessory protein AcrZ | 2.86 |
| dinI^a^ | b1061 | mRNA | cytosol | DNA damage-inducible protein I | 2.84 |
| ybfA^a^ | b0699 | mRNA | inner membrane, cytosol | DUF2517 domain-containing protein YbfA | 2.78 |
| dsdX | b2365 | mRNA | inner membrane | D-serine transporter | 2.73 |
| allR^a^ | b0506 | mRNA | cytosol | DNA-binding transcriptional repressor AllR | 2.45 |
| yedK | b1931 | mRNA | cytosol | genome maintenance protein | 2.35 |
| bglJ | b4366 | mRNA | cytosol | DNA-binding transcriptional regulator BglJ | 2.22 |
| bsmA | b4189 | mRNA | inner membrane, periplasmic space | DUF1471 domain-containing putative lipoprotein BsmA | 2.20 |
| sulA^a^ | b0958 | mRNA | inner membrane | cell division inhibitor SulA | 2.16 |
| mgtL^a^ | b4702 | mRNA | cytosol | leader peptide MgtL | 2.10 |
| baeS | b2078 | mRNA | inner membrane | sensor histidine kinase BaeS | 2.07 |
| ytfK^a^ | b4217 | mRNA | cytosol | stringent response modulator YtfK | 2.06 |
| znuB | b1859 | mRNA | inner membrane | Zn2+ABC transporter membrane subunit | 2.02 |
| sanA | b2144 | mRNA | periplasmic space, inner membrane | DUF218 domain-containing protein SanA | 2.01 |
| **anaerobic respiration** | |  |  |  |  |
| nirD | b3366 | mRNA | cytosol | nitrite reductase subunit NirD | 10.38 |
| torZ | b1872 | mRNA | periplasmic space | trimethylamineN-oxide reductase 2 | 2.10 |
| **biosynthetic process** | |  |  |  |  |
| hisL^a^ | b2018 | mRNA | cytosol | hisoperon leader peptide | 5.89 |
| ilvL^a^ | b3766 | mRNA | cytosol | ilvXGMEDAoperon leader peptide | 5.46 |
| trpE | b1264 | mRNA | cytosol | anthranilate synthase subunit TrpE | 4.99 |
| ccmD | b2198 | mRNA | inner membrane | cytochromecmaturation protein D | 3.93 |
| yhbV^a^ | b3159 | mRNA | cytosol | ubiquinone biosynthesis protein UbiV | 3.84 |
| trpL | b1265 | mRNA | membrane | trpoperon leader peptide | 3.76 |
| leuD^a^ | b0071 | mRNA | cytosol | 3-isopropylmalate dehydratase subunit LeuD | 3.01 |
| leuL^a^ | b0075 | mRNA | cytosol | leuoperon leader peptide | 2.97 |
| cof^a^ | b0446 | mRNA | cytosol | HMP-PP phosphatase | 2.63 |
| coaD^a^ | b3634 | mRNA | cytosol | pantetheine-phosphate adenylyltransferase | 2.58 |
| **catabolic process** | |  |  |  |  |
| eutA | b2451 | mRNA | inner membrane | ethanolamine ammonia-lyase reactivase EutA | 9.76 |
| idnO | b4266 | mRNA | cytosol | 5-keto-D-gluconate 5-reductase | 7.80 |
| tdcD | b3115 | mRNA | cytosol | propionate kinase | 4.82 |
| allB^a^ | b0512 | mRNA | cytosol | allantoinase | 3.81 |
| ycaL | b0909 | mRNA | inner membrane, periplasmic space, extracellular space | periplasmic protease YcaL | 3.57 |
| dgoD^a^ | b4478 | mRNA | cytosol | D-galactonate dehydratase | 2.98 |
| tdcF | b3113 | mRNA | cytosol | predicted enamine/imine deaminase | 2.76 |
| xylA | b3565 | mRNA | cytosol | xylose isomerase | 2.72 |
| fucI^a^ | b2802 | mRNA | cytosol | L-fucose isomerase | 2.54 |
| garR | b3125 | mRNA | cytosol | tartronate semialdehyde reductase | 2.50 |
| chiA^a^ | b3338 | mRNA | extracellular space, periplasmic space | endochitinase | 2.50 |
| astD | b1746 | mRNA | cytosol | aldehyde dehydrogenase | 2.24 |
| chbG | b1733 | mRNA | cytosol | chitin disaccharide deacetylase | 2.11 |
| **regulation of single-species biofilm formation** | | | | | |
| ryfD^a^ | b4609 | ncRNA | no annotation | small regulatory RNA RyfD | 32.24 |
| dsrA^a^ | b1954 | ncRNA | no annotation | small regulatory RNA DsrA | 4.42 |
| csrC^a^ | b4457 | ncRNA | no annotation | small regulatory RNA CsrC | 2.97 |
| ycfJ | b1110 | mRNA | inner membrane | PF05433 family protein YcfJ | 2.76 |
| **lipid metabolic process** | | |  |  |  |
| fepE | b0587 | mRNA | inner membrane | polysaccharide co-polymerase family protein FepE | 9.46 |
| arnF | b2258 | mRNA | inner membrane | undecaprenyl-phosphate-α-L-Ara4N flippase - ArnF subunit | 3.18 |
| ybgC | b0736 | mRNA | inner membrane, cytosol | esterase/thioesterase | 2.57 |
| acpP | b1094 | mRNA | cytosol | acyl carrier protein | 2.38 |
| etk | b0981 | mRNA | inner membrane | protein-tyrosine kinase Etk | 2.31 |
| lapA^a^ | b1279 | mRNA | inner membrane | lipopolysaccharide assembly protein A | 2.26 |
| lpp^a^ | b1677 | mRNA | outer membrane, cell wall, extracellular space, periplasmic space | murein lipoprotein | 2.20 |
| **cell adhesion** | |  |  |  |  |
| csgA | b1042 | mRNA | extracellular space, pilus | curlin, major subunit | 7.04 |
| sfmF^a^ | b0534 | mRNA | extracellular space, pilus | putative fimbrial protein SfmF | 3.95 |
| dgcF | b1522 | mRNA | inner membrane | putative diguanylate cyclase DgcF | 2.86 |
| **DNA recombination** | |  |  |  |  |
| fimE^a^ | b4313 | mRNA | cytosol | regulator for fimA | 2.92 |
| insQ^a^ | b1432 | mRNA | cytosol | putative insertion element transposase InsQ | 2.33 |
| sbcD^a^ | b0398 | mRNA | cytosol | ATP-dependent structure-specific DNA nuclease - SbcD subunit | 2.19 |
| **translation** |  |  |  |  |  |
| fnrS^a^ | b4699 | ncRNA | no annotation | small regulatory RNA FnrS | 11.35 |
| glmZ^a^ | b4456 | ncRNA | no annotation | small regulatory RNA GlmZ | 7.00 |
| istR^a^ | b4616 | ncRNA | no annotation | small regulatory RNA IstR-1 | 6.29 |
| spf^a^ | b3864 | ncRNA | no annotation | small regulatory RNA Spot 42 | 3.72 |
| omrB^a^ | b4445 | ncRNA | no annotation | small regulatory RNA OmrB | 3.71 |
| mgrR^a^ | b4698 | ncRNA | no annotation | small regulatory RNA MgrR | 3.12 |
| gcvB^a^ | b4443 | ncRNA | no annotation | small regulatory RNA GcvB | 3.09 |
| **transport** |  |  |  |  |  |
| xylG^a^ | b3567 | mRNA | inner membrane | xylose ABC transporter ATP binding subunit | 4.53 |
| citT | b0612 | mRNA | inner membrane | citrate:succinate antiporter | 4.51 |
| ydeA | b1528 | mRNA | inner membrane | L-arabinose exporter | 3.02 |
| araE^a^ | b2841 | mRNA | inner membrane | arabinose:H+symporter | 2.86 |
| ycaD^a^ | b0898 | mRNA | inner membrane | putative transporter YcaD | 2.66 |
| dauA^a^ | b1206 | mRNA | inner membrane | aerobic C4-dicarboxylate transporter DauA | 2.43 |
| sfmD | b0532 | mRNA | outer membrane | putative fimbrial usher protein SfmD | 2.41 |
| fieF^a^ | b3915 | mRNA | inner membrane | Zn2+/Fe2+/Cd2+exporter | 2.29 |
| ytfR | b4485 | mRNA | inner membrane | galactofuranose ABC transporter putative ATP binding subunit | 2.25 |
| yfeO^a^ | b2389 | mRNA | inner membrane | putative transport protein YfeO | 2.19 |
| araH | b4460 | mRNA | inner membrane | arabinose ABC transporter membrane subunit | 2.10 |
| yihN | b3874 | mRNA | inner membrane | putative transporter YihN | 2.09 |
| eamB^a^ | b2578 | mRNA | inner membrane | cysteine/O-acetylserine exporter EamB | 2.08 |
| xapB | b2406 | mRNA | inner membrane | xanthosine:H+symporter XapB | 2.07 |
| yjbB | b4020 | mRNA | inner membrane | putative inorganic phosphate export protein YjbB | 2.02 |
| **rRNA** |  |  |  |  |  |
| rrfF^a^ | b3272 | rRNA | cytosol | 5S ribosomal RNA | 46.07 |
| rrfB^a^ | b3971 | rRNA | cytosol | 5S ribosomal RNA | 44.28 |
| rrfE^a^ | b4010 | rRNA | cytosol | 5S ribosomal RNA | 42.11 |
| rrfG^a^ | b2588 | rRNA | cytosol | 5S ribosomal RNA | 36.00 |
| rrfH^a^ | b0205 | rRNA | cytosol | 5S ribosomal RNA | 35.27 |
| rrfD^a^ | b3274 | rRNA | cytosol | 5S ribosomal RNA | 34.85 |
| rrfC^a^ | b3759 | rRNA | cytosol | 5S ribosomal RNA | 30.58 |
| rrfA^a^ | b3855 | rRNA | cytosol | 5S ribosomal RNA | 12.07 |
| **tRNA** |  |  |  |  |  |
| trpT^a^ | b3761 | tRNA | cytosol | tRNA-Trp(CCA) | 13.02 |
| leuU^a^ | b3174 | tRNA | cytosol | tRNA-Leu(GAG) | 12.13 |
| alaW^a^ | b2397 | tRNA | cytosol | tRNA-Ala(GGC) | 10.81 |
| glyT^a^ | b3978 | tRNA | cytosol | tRNA-Gly(UCC) | 10.74 |
| valU^a^ | b2401 | tRNA | cytosol | tRNA-Val(UAC) | 6.92 |
| selC^a^ | b3658 | tRNA | cytosol | tRNA-Sec(UCA) | 5.52 |
| leuQ^a^ | b4370 | tRNA | cytosol | tRNA-Leu(CAG) | 5.11 |
| gltU^a^ | b3757 | tRNA | cytosol | tRNA-Glu(UUC) | 4.83 |
| metZ^a^ | b2814 | tRNA | cytosol | tRNA-initiator Met(CAU) | 4.49 |
| serU^a^ | b1975 | tRNA | cytosol | tRNA-Ser(CGA) | 4.48 |
| leuX^a^ | b4270 | tRNA | cytosol | tRNA-Leu(CAA) | 4.11 |
| gltV^a^ | b4008 | tRNA | cytosol | tRNA-Glu(UUC) | 3.87 |
| aspU^a^ | b0206 | tRNA | cytosol | tRNA-Asp(GUC) | 3.85 |
| ileV^a^ | b0202 | tRNA | cytosol | tRNA-Ile(GAU) | 3.80 |
| leuZ^a^ | b1909 | tRNA | cytosol | tRNA-Leu(UAA) | 3.80 |
| argX^a^ | b3796 | tRNA | cytosol | tRNA-Arg(CCG) | 3.68 |
| serV^a^ | b2695 | tRNA | cytosol | tRNA-Ser(GCU) | 3.58 |
| aspV^a^ | b0216 | tRNA | cytosol | tRNA-Asp(GUC) | 3.52 |
| ileU^a^ | b3277 | tRNA | cytosol | tRNA-Ile(GAU) | 3.49 |
| ileT^a^ | b3852 | tRNA | cytosol | tRNA-Ile(GAU) | 3.20 |
| metY^a^ | b3171 | tRNA | cytosol | tRNA-initiator Met(CAU) | 3.11 |
| metV^a^ | b2816 | tRNA | cytosol | tRNA-initiator Met(CAU) | 2.85 |
| metW^a^ | b2815 | tRNA | cytosol | tRNA-initiator Met(CAU) | 2.27 |
| argQ | b2691 | tRNA | cytosol | tRNA-Arg(ACG) | 2.22 |
| alaU^a^ | b3276 | tRNA | cytosol | tRNA-Ala(UGC) | 2.21 |
| alaT | b3853 | tRNA | cytosol | tRNA-Ala(UGC) | 2.20 |
| **other** |  |  |  |  |  |
| sraG | b4449 | ncRNA | no annotation | small regulatory RNA SraG | 17.73 |
| ffs^a^ | b0455 | ncRNA | cytosol | signal recognition particle 4.5S RNA | 17.33 |
| flhE | b1878 | mRNA | periplasmic space, cell projection | flagellar protein FlhE | 7.55 |
| sgcX | b4305 | mRNA | cytosol | KpLE2 phage-like element; putative endoglucanase with Zn-dependent exopeptidase domain | 6.06 |
| trmO^a^ | b0195 | mRNA | cytosol | tRNA m6t6A37 methyltransferase | 4.50 |
| csgG^a^ | b1037 | mRNA | periplasmic space, inner membrane, outer membrane | curli secretion channel | 4.09 |
| gspO | b3335 | mRNA | inner membrane | Type II secretion system prepilin peptidase | 3.95 |
| yjfN | b4188 | mRNA | periplasmic space | protease activator YjfN | 3.83 |
| insA-3^a^ | b0275 | mRNA | cytosol | IS1 protein InsA | 3.62 |
| hypA | b2726 | mRNA | inner membrane, cytosol | hydrogenase 3 nickel incorporation protein HypA | 3.41 |
| cutC^a^ | b1874 | mRNA | cytosol | protein CutC | 2.97 |
| ygfS^a^ | b2886 | mRNA | inner membrane | putative electron transport protein YgfS | 2.85 |
| ygcE | b2776 | mRNA | cytosol | putative sugar kinase YgcE | 2.56 |
| malM | b4037 | mRNA | periplasmic space | maltose regulon periplasmic protein | 2.48 |
| gluQ^a^ | b0144 | mRNA | cytosol | glutamyl-Q tRNAAspsynthetase | 2.39 |
| nlpC^a^ | b1708 | mRNA | inner membrane, periplasmic space | NlpC/P60 family lipoprotein NlpC | 2.33 |
| ftsL^a^ | b0083 | mRNA | inner membrane | cell division protein FtsL | 2.28 |
| rnd^a^ | b1804 | mRNA | cytosol | RNase D | 2.22 |
| hybE^a^ | b2992 | mRNA | cytosol | hydrogenase 2-specific chaperone | 2.16 |
| smf^a^ | b4473 | mRNA | cytosol | protein Smf | 2.16 |
| hypD^a^ | b2729 | mRNA | cytosol | Fe-(CN)2CO cofactor assembly scaffold protein HypD | 2.09 |
| glpE | b3425 | mRNA | cytosol | thiosulfate sulfurtransferase GlpE | 2.04 |
| pspG | b4050 | mRNA | inner membrane | phage shock protein G | 2.02 |
| ryjA^a^ | b4459 | ncRNA | no annotation | small RNA RyjA | 12.34 |
| yjiG | b4329 | mRNA | inner membrane | Gate family protein YjiG | 10.16 |
| ssrS^a^ | b2911 | ncRNA | no annotation | 6S RNA | 9.97 |
| ymfH | b1142 | mRNA | inner membrane | e14 prophage; putative protein YmfH | 9.37 |
| ychQ^a^ | b1213 | mRNA | inner membrane | SirB family protein YchQ | 7.25 |
| ykgR^a^ | b4671 | mRNA | inner membrane | uncharacterized membrane protein YkgR | 7.04 |
| ralA | b4714 | ncRNA | no annotation | Rac prophage; small regulatory RNA antitoxin RalA | 6.59 |
| sroH^a^ | b4691 | ncRNA | no annotation | small RNA SroH | 5.55 |
| ryeA^a^ | b4432 | ncRNA | no annotation | small antisense RNA RyeA | 5.26 |
| yfaP | b2225 | mRNA | cytosol | DUF2135 domain-containing protein YfaP | 4.47 |
| atoE | b2223 | mRNA | inner membrane | short chain fatty acid transporter | 4.21 |
| yncL^a^ | b4598 | mRNA | inner membrane | uncharacterized protein YncL | 4.16 |
| yfiM^a^ | b2586 | mRNA | inner membrane | protein YfiM | 3.94 |
| ypdK^a^ | b4680 | mRNA | inner membrane | uncharacterized membrane protein YpdK | 3.83 |
| yohP^a^ | b4679 | mRNA | inner membrane | uncharacterized membrane protein YohP | 3.57 |
| ryjB^a^ | b4624 | ncRNA | no annotation | small RNA RyjB | 3.02 |
| yohO | b4542 | mRNA | inner membrane | UPF0387 family protein YohO | 2.98 |
| yecH^a^ | b1906 | mRNA | cytosol | DUF2492 domain-containing protein YecH | 2.96 |
| yciY^a^ | b4595 | mRNA | cytosol | uncharacterized protein YciY | 2.89 |
| sraB^a^ | b4418 | ncRNA | no annotation | small RNA SraB | 2.88 |
| yebB^a^ | b1862 | mRNA | cytosol | putative papain-like amidase YebB | 2.75 |
| yddH | b1462 | mRNA | cytosol | flavin reductase-like protein YddH | 2.61 |
| gnsA | b4517 | mRNA | cytosol | putative phosphatidylethanolamine synthesis regulator GnsA | 2.60 |
| ybiW | b0823 | mRNA | cytosol | putative pyruvate formate lyase | 2.53 |
| ytfF^a^ | b4210 | mRNA | inner membrane | inner membrane protein YtfF | 2.46 |
| ycaR^a^ | b0917 | mRNA | cytosol | PF03966 family protein YcaR | 2.46 |
| yzgL | b3427 | mRNA | membrane | putative uncharacterized protein YzgL | 2.43 |
| yidB | b3698 | mRNA | cytosol | DUF937 domain-containing protein YidB | 2.38 |
| dsrB^a^ | b1952 | mRNA | cytosol | protein DsrB | 2.34 |
| insA9 | b4709 | mRNA | cytosol | IS1 repressor TnpA | 2.32 |
| yobH | b4536 | mRNA | inner membrane | uncharacterized protein YobH | 2.30 |
| yniD^a^ | b4535 | mRNA | inner membrane | uncharacterized protein YniD | 2.29 |
| ysdD | b4757 | mRNA | cytosol | protein YsdD | 2.29 |
| fumD^a^ | b1675 | mRNA | cytosol | fumarase D | 2.28 |
| rdlA | b4420 | ncRNA | no annotation | putative antisense regulatory RNA RdlA | 2.28 |
| ynjD | b1756 | mRNA | inner membrane | putative ABC transporter ATP-binding protein YnjD | 2.27 |
| tfaP^a^ | b1155 | mRNA | cytosol | e14 prophage; putative tail fiber assembly protein TfaP | 2.23 |
| yhdV | b3267 | mRNA | inner membrane | lipoprotein YhdV | 2.22 |
| rem | b1561 | mRNA | cytosol | Qin prophage; protein Rem | 2.20 |
| yecF^a^ | b1915 | mRNA | cytosol | DUF2594 domain-containing protein YecF | 2.10 |
| gltF | b3214 | mRNA | inner membrane, periplasmic space | periplasmic protein GltF | 2.09 |
| azuC^a^ | b4663 | mRNA | inner membrane | uncharacterized protein AzuC | 2.09 |
| ytjA | b4568 | mRNA | inner membrane | DUF1328 domain-containing protein YtjA | 2.04 |
