# Supplementary material for: Genome-wide identification of Kanamycin B binding RNA in Escherichia coli
Source: BMC Genomics. 2023 Mar 16;24:120. doi: 10.1186/s12864-023-09234-3 (PMC10018874; doi:10.1186/s12864-023-09234-3)
Supplement: Supplementary file 2 — Additional file 2: Table S2. Fold change and gene functions of 230 enrichment genes in 1 μM Kanamycin B pull down assay. [file 12864_2023_9234_MOESM2_ESM.docx]

**Table S2. Fold change and gene functions of 230 enrichment genes in 1μM Kanamycin B pull down assay.**

^a^ 134 overlapped enrichment genes in 0μM Kanamycin B and 1μM Kanamycin B pull down assay.

| **Transcript Name** | **Transcript ID** | | **Type** | **Location** | **Product** | | | **Fold Change** |
| --- | --- | --- | --- | --- | --- | --- | --- | --- |
| **Cell death** | |  | | | |  |  |  |
| ibsC^a^ | b4665 | | mRNA | inner membrane | toxic peptide IbsC | | | 60.49 |
| ibsD^a^ | b4664 | | mRNA | inner membrane | putative toxic peptide IbsD | | | 24.27 |
| ldrA^a^ | b4419 | | mRNA | inner membrane | small toxic polypeptide LdrA | | | 5.02 |
| shoB^a^ | b4687 | | mRNA | inner membrane | toxic peptide ShoB | | | 4.92 |
| ldrC^a^ | b4423 | | mRNA | inner membrane | small toxic polypeptide LdrC | | | 3.23 |
| hokD | b1562 | | mRNA | inner membrane | Qin prophage; toxic protein HokD | | | 2.34 |
| **post-transcriptional gene silencing by RNA** | | | | | | | | |
| sibD^a^ | b4447 | | ncRNA | no annotation | small RNA SibD | | | 14.08 |
| sibC^a^ | b4446 | | ncRNA | no annotation | small regulatory RNA antitoxin SibC | | | 5.04 |
| sibE^a^ | b4611 | | ncRNA | no annotation | small RNA SibE | | | 4.90 |
| sibB^a^ | b4437 | | ncRNA | no annotation | small RNA SibB | | | 4.34 |
| sibA^a^ | b4436 | | ncRNA | no annotation | small RNA SibA | | | 2.52 |
| **transcription, DNA-templated** | | | | | | | |  |
| lrhA^a^ | b2289 | | mRNA | cytosol | DNA-binding transcriptional dual regulator LrhA | | | 6.44 |
| pyrL^a^ | b4246 | | mRNA | cytosol | pyrBIoperon leader peptide | | | 3.59 |
| ybeF | b0629 | | mRNA | cytosol | putative LysR-type DNA-binding transcriptional regulator YbeF | | | 2.42 |
| sutR | b1434 | | mRNA | cytosol | DNA-binding transcriptional dual regulator SutR | | | 2.39 |
| cecR^a^ | b0796 | | mRNA | cytosol | DNA-binding transcriptional dual regulator CecR | | | 2.21 |
| nusG | b3982 | | mRNA | cytosol | transcription termination/antitermination factor NusG | | | 2.20 |
| thrL^a^ | b0001 | | mRNA | cytosol | throperon leader peptide | | | 2.14 |
| hupB^a^ | b0440 | | mRNA | cytosol | DNA-binding protein HU-β | | | 2.07 |
| tnaC | b3707 | | mRNA | cytosol | tnaABoperon leader peptide | | | 2.07 |
| **response to stimulus** | | | | | | | | |
| glmY^a^ | b4441 | | ncRNA |  | small regulatory RNA GlmY | | | 17.82 |
| ves | b1742 | | mRNA | cytosol | HutD family protein Ves | | | 11.09 |
| ynhF^a^ | b4602 | | mRNA | inner membrane | cytochromebd-I ubiquinol oxidase accessory subunit CydH | | | 10.05 |
| glxR | b0509 | | mRNA | cytosol | tartronate semialdehyde reductase 2 | | | 5.83 |
| cusC | b0572 | | mRNA | outer membrane | copper/silver export system outer membrane channel | | | 4.55 |
| ftsI^a^ | b0084 | | mRNA | inner membrane | peptidoglycan DD-transpeptidase FtsI | | | 4.22 |
| acrZ^a^ | b0762 | | mRNA | cytosol, outer membrane, inner membrane | multidrug efflux pump accessory protein AcrZ | | | 3.68 |
| allR^a^ | b0506 | | mRNA | cytosol | DNA-binding transcriptional repressor AllR | | | 3.27 |
| rmf^a^ | b0953 | | mRNA | cytosol | ribosome modulation factor | | | 3.20 |
| dinI^a^ | b1061 | | mRNA | cytosol | DNA damage-inducible protein I | | | 3.20 |
| dinQ^a^ | b4613 | | mRNA | inner membrane | membrane toxin DinQ | | | 3.11 |
| ybfA^a^ | b0699 | | mRNA | inner membrane, cytosol | DUF2517 domain-containing protein YbfA | | | 2.97 |
| ecnB^a^ | b4411 | | mRNA | inner membrane, outer membrane | bacteriolytic entericidin B lipoprotein | | | 2.85 |
| mgtS^a^ | b4599 | | mRNA | inner membrane | small protein MgtS | | | 2.78 |
| yqaE^a^ | b2666 | | mRNA | inner membrane | Pmp3 family protein YqaE | | | 2.72 |
| rpsQ | b3311 | | mRNA | ribosome, cytosol | 30S ribosomal subunit protein S17 | | | 2.49 |
| yidH | b3676 | | mRNA | inner membrane | DUF202 domain-containing inner membrane protein YidH | | | 2.42 |
| yphB^a^ | b2544 | | mRNA | cytosol | putative aldose 1-epimerase YphB | | | 2.33 |
| dusB | b3260 | | mRNA | cytosol | tRNA-dihydrouridine synthase B | | | 2.28 |
| mgtL^a^ | b4702 | | mRNA | cytosol | leader peptide MgtL | | | 2.24 |
| recO | b2565 | | mRNA | bacterial nucleoid, cytosol | DNA repair protein RecO | | | 2.19 |
| oxyS^a^ | b4458 | | ncRNA | bacterial nucleoid, cytosol | small regulatory RNA OxyS | | | 2.15 |
| gshA | b2688 | | mRNA | cytosol | glutamate—cysteine ligase | | | 2.12 |
| dolP | b3150 | | mRNA | periplasmic space, outer membrane | division and outer membrane stress-associated lipid-binding lipoprotein | | | 2.11 |
| rseC | b2570 | | mRNA | inner membrane | protein RseC | | | 2.10 |
| ytfK^a^ | b4217 | | mRNA | cytosol | stringent response modulator YtfK | | | 2.05 |
| sulA^a^ | b0958 | | mRNA | inner membrane | cell division inhibitor SulA | | | 2.14 |
| narQ | b2469 | | mRNA | inner membrane | sensory histidine kinase NarQ | | | 2.04 |
| **biosynthetic process** | | | | | | | |  |
| hisL^a^ | b2018 | | mRNA | cytosol | hisoperon leader peptide | | | 7.99 |
| yiaT | b3584 | | mRNA | outer membrane | outer membrane protein YiaT | | | 5.95 |
| bioD | b0778 | | mRNA | cytosol | dethiobiotin synthetase | | | 5.31 |
| ilvL^a^ | b3766 | | mRNA | cytosol | ilvXGMEDAoperon leader peptide | | | 4.26 |
| leuL^a^ | b0075 | | mRNA | cytosol | leuoperon leader peptide | | | 3.86 |
| ulaD | b4196 | | mRNA | cytosol | 3-keto-L-gulonate-6-phosphate decarboxylase UlaD | | | 3.85 |
| entD | b0583 | | mRNA | inner membrane, cytosol | phosphopantetheinyl transferase EntD | | | 3.41 |
| cof^a^ | b0446 | | mRNA | cytosol | HMP-PP phosphatase | | | 2.88 |
| leuD^a^ | b0071 | | mRNA | cytosol | 3-isopropylmalate dehydratase subunit LeuD | | | 2.70 |
| yhbV^a^ | b3159 | | mRNA | cytosol | ubiquinone biosynthesis protein UbiV | | | 2.69 |
| trpD | b1263 | | mRNA | cytosol | anthranilate synthase subunit TrpD | | | 2.26 |
| atpC | b3731 | | mRNA | inner membrane | ATP synthase F1complex subunit ε | | | 2.25 |
| coaD^a^ | b3634 | | mRNA | cytosol | pantetheine-phosphate adenylyltransferase | | | 2.15 |
| ribB | b3041 | | mRNA | inner membrane, cytosol | 3,4-dihydroxy-2-butanone-4-phosphate synthase | | | 2.12 |
| bioH | b3412 | | mRNA | cytosol | pimeloyl-acyl carrier protein methyl ester esterase | | | 2.06 |
| **catabolic process** | | | | |  | | |  |
| idnK | b4268 | | mRNA | cytosol | D-gluconate kinase, thermosensitive | | | 8.37 |
| sgbE | b3583 | | mRNA | cytosol | L-ribulose-5-phosphate 4-epimerase SgbE | | | 7.13 |
| idnT | b4265 | | mRNA | inner membrane | L-idonate/5-ketogluconate/gluconate transporter | | | 4.55 |
| gntP | b4321 | | mRNA | inner membrane | fructuronate transporter | | | 3.74 |
| phnP | b4092 | | mRNA | cytosol | 5-phospho-α-D-ribosyl 1,2-cyclic phosphate phosphodiesterase | | | 3.36 |
| allB^a^ | b0512 | | mRNA | cytosol | allantoinase | | | 3.12 |
| dgoD^a^ | b4478 | | mRNA | cytosol | D-galactonate dehydratase | | | 3.10 |
| fucI^a^ | b2802 | | mRNA | cytosol | L-fucose isomerase | | | 2.70 |
| aegA | b2468 | | mRNA | cytosol | putative oxidoreductase AegA | | | 2.16 |
| chiA^a^ | b3338 | | mRNA | extracellular space, periplasmic space | endochitinase | | | 2.05 |
| **regulation of single-species biofilm formation** | | | | | | | | |
| ryfD^a^ | b4609 | | ncRNA | no annotation | small regulatory RNA RyfD | | | 45.91 |
| dicF | b1574 | | ncRNA | no annotation | Qin prophage; small regulatory RNA DicF | | | 16.18 |
| arcZ | b4450 | | ncRNA | no annotation | small regulatory RNA ArcZ | | | 7.71 |
| dsrA^a^ | b1954 | | ncRNA | no annotation | small regulatory RNA DsrA | | | 3.98 |
| csrC^a^ | b4457 | | ncRNA | no annotation | small regulatory RNA CsrC | | | 2.13 |
| **lipid metabolic process** | | | | | | | |  |
| lpp^a^ | b1677 | | mRNA | outer membrane, cell wall, extracellular space, periplasmic space | murein lipoprotein | | | 2.57 |
| lapA^a^ | b1279 | | mRNA | inner membrane | lipopolysaccharide assembly protein A | | | 2.31 |
| **oxidation-reduction process** | | | | | | | | |
| ydiS | b1699 | | mRNA | cytosol | putative electron transfer flavoprotein-quinone oxidoreductase YdiS | | | 6.69 |
| torA | b0997 | | mRNA | periplasmic space | trimethylamineN-oxide reductase 1 | | | 3.07 |
| rsxG | b1631 | | mRNA | inner membrane, outer membrane | SoxR [2Fe-2S] reducing system protein RsxG | | | 3.03 |
| ygfS^a^ | b2886 | | mRNA | inner membrane | putative electron transport protein YgfS | | | 2.93 |
| napH | b2204 | | mRNA | inner membrane | ferredoxin-type protein NapH | | | 2.53 |
| yceM | b1068 | | mRNA | cytosol | putative oxidoreductase YceM | | | 2.11 |
| ccmH | b2194 | | mRNA | inner membrane | holocytochromecsynthase CcmH component | | | 2.10 |
| **DNA recombination** | | | |  |  | | |  |
| insQ^a^ | b1432 | | mRNA | cytosol | putative insertion element transposase InsQ | | | 2.39 |
| sbcD^a^ | b0398 | | mRNA | cytosol | ATP-dependent structure-specific DNA nuclease - SbcD subunit | | | 2.22 |
| fimE^a^ | b4313 | | mRNA | cytosol | regulator for fimA | | | 2.08 |
| **translation** | | | |  |  | | |  |
| fnrS^a^ | b4699 | | ncRNA | no annotation | small regulatory RNA FnrS | | | 11.32 |
| glmZ^a^ | b4456 | | ncRNA | no annotation | small regulatory RNA GlmZ | | | 7.34 |
| spf^a^ | b3864 | | ncRNA | no annotation | small regulatory RNA Spot 42 | | | 4.43 |
| omrB^a^ | b4445 | | ncRNA | no annotation | small regulatory RNA OmrB | | | 4.27 |
| gcvB^a^ | b4443 | | ncRNA | no annotation | small regulatory RNA GcvB | | | 3.87 |
| istR^a^ | b4616 | | ncRNA | no annotation | small regulatory RNA IstR-1 | | | 3.37 |
| rpsI | b3230 | | mRNA | ribosome, cytosol | 30S ribosomal subunit protein S9 | | | 2.52 |
| rplI | b4203 | | mRNA | ribosome, cytosol | 50S ribosomal subunit protein L9 | | | 2.40 |
| mgrR^a^ | b4698 | | ncRNA | no annotation | small regulatory RNA MgrR | | | 2.09 |
| **regulation of cell shape** | | | |  |  | | |  |
| mreD | b3249 | | mRNA | inner membrane | cell shape determining protein MreD | | | 2.76 |
| dacB | b3182 | | mRNA | periplasmic space, inner membrane | peptidoglycan DD endopeptidase DacB | | | 2.15 |
| ybjG | b0841 | | mRNA | inner membrane | undecaprenyl pyrophosphate phosphatase | | | 2.06 |
| **regulation of bacterial-type flagellum-dependent cell motility** | | | | | | | | |
| fliG | b1939 | | mRNA | inner membrane, cell projection | flagellar motor switch protein FliG | | | 12.73 |
| flgB | b1073 | | mRNA | periplasmic space, cell projection | flagellar basal-body rod protein FlgB | | | 9.06 |
| flgA | b1072 | | mRNA | periplasmic space | flagellar basal body P-ring formation protein FlgA | | | 3.81 |
| **transport** | | |  |  |  | | |  |
| yhdX | b3269 | | mRNA | inner membrane | putative ABC transporter membrane subunit YhdX | | | 5.85 |
| lysO | b0874 | | mRNA | inner membrane | L-lysine exporter | | | 3.82 |
| mdtI | b1599 | | mRNA | inner membrane | multidrug/spermidine efflux pump membrane subunit MdtI | | | 3.45 |
| ynjC | b1755 | | mRNA | inner membrane | putative ABC transporter membrane subunit YnjC | | | 3.14 |
| fieF^a^ | b3915 | | mRNA | inner membrane | Zn2+/Fe2+/Cd2+exporter | | | 3.13 |
| chbC | b1737 | | mRNA | inner membrane | N,N'-diacetylchitobiose-specific PTS enzyme IIC component | | | 2.98 |
| ybbY | b0513 | | mRNA | inner membrane | putative purine transporter | | | 2.86 |
| fryA | b2383 | | mRNA | cytosol, membrane | putative PTS multiphosphoryl transfer protein FryA | | | 2.84 |
| araE^a^ | b2841 | | mRNA | inner membrane | arabinose:H+symporter | | | 2.69 |
| xylG^a^ | b3567 | | mRNA | inner membrane | xylose ABC transporter ATP binding subunit | | | 2.35 |
| psuT | b2164 | | mRNA | inner membrane | putative pseudouridine transporter | | | 2.28 |
| gntU | b4476 | | mRNA | inner membrane | low-affinity gluconate transporter | | | 2.26 |
| ycaD^a^ | b0898 | | mRNA | inner membrane | putative transporter YcaD | | | 2.25 |
| ompW | b1256 | | mRNA | outer membrane | outer membrane protein W | | | 2.25 |
| dauA^a^ | b1206 | | mRNA | inner membrane | aerobic C4-dicarboxylate transporter DauA | | | 2.24 |
| eamB^a^ | b2578 | | mRNA | inner membrane | cysteine/O-acetylserine exporter EamB | | | 2.22 |
| tqsA | b1601 | | mRNA | inner membrane | autoinducer 2 exporter | | | 2.19 |
| ygbN | b2740 | | mRNA | inner membrane | putative transporter YgbN | | | 2.15 |
| potE | b0692 | | mRNA | inner membrane | putrescine transporter PotE | | | 2.13 |
| btuD | b1709 | | mRNA | inner membrane | vitamin B12ABC transporter ATP binding subunit | | | 2.11 |
| yfeO^a^ | b2389 | | mRNA | inner membrane | putative transport protein YfeO | | | 2.08 |
| pheP | b0576 | | mRNA | inner membrane | phenylalanine:H+symporter PheP | | | 2.06 |
| btuC | b1711 | | mRNA | inner membrane | vitamin B12ABC transporter membrane subunit | | | 2.01 |
| **rRNA** |  | |  |  |  | | |  |
| rrfF^a^ | b3272 | | rRNA | cytosol | 5S ribosomal RNA | | | 61.87 |
| rrfB^a^ | b3971 | | rRNA | cytosol | 5S ribosomal RNA | | | 56.83 |
| rrfE^a^ | b4010 | | rRNA | cytosol | 5S ribosomal RNA | | | 56.12 |
| rrfG^a^ | b2588 | | rRNA | cytosol | 5S ribosomal RNA | | | 48.15 |
| rrfD^a^ | b3274 | | rRNA | cytosol | 5S ribosomal RNA | | | 45.71 |
| rrfH^a^ | b0205 | | rRNA | cytosol | 5S ribosomal RNA | | | 45.14 |
| rrfC^a^ | b3759 | | rRNA | cytosol | 5S ribosomal RNA | | | 38.82 |
| rrfA^a^ | b3855 | | rRNA | no annotation | 5S ribosomal RNA | | | 18.27 |
| **tRNA** |  | |  |  |  | | |  |
| trpT^a^ | b3761 | | tRNA | cytosol | tRNA-Trp(CCA) | | | 49.42 |
| alaW^a^ | b2397 | | tRNA | cytosol | tRNA-Ala(GGC) | | | 15.94 |
| valV | b1665 | | tRNA | cytosol | tRNA-Val(GAC) | | | 10.53 |
| selC^a^ | b3658 | | tRNA | cytosol | tRNA-Sec(UCA) | | | 9.63 |
| valU^a^ | b2401 | | tRNA | cytosol | tRNA-Val(UAC) | | | 8.60 |
| leuU^a^ | b3174 | | tRNA | cytosol | tRNA-Leu(GAG) | | | 7.92 |
| glnX | b0664 | | tRNA | cytosol | tRNA-Gln(CUG) | | | 7.44 |
| aspU^a^ | b0206 | | tRNA | cytosol | tRNA-Asp(GUC) | | | 7.34 |
| gltU^a^ | b3757 | | tRNA | cytosol | tRNA-Glu(UUC) | | | 6.94 |
| leuZ^a^ | b1909 | | tRNA | cytosol | tRNA-Leu(UAA) | | | 6.19 |
| glyT^a^ | b3978 | | tRNA | cytosol | tRNA-Gly(UCC) | | | 5.97 |
| leuQ^a^ | b4370 | | tRNA | cytosol | tRNA-Leu(CAG) | | | 5.23 |
| gltV^a^ | b4008 | | tRNA | cytosol | tRNA-Glu(UUC) | | | 4.92 |
| metV^a^ | b2816 | | tRNA | cytosol | tRNA-initiator Met(CAU) | | | 4.62 |
| serV^a^ | b2695 | | tRNA | cytosol | tRNA-Ser(GCU) | | | 4.29 |
| metZ^a^ | b2814 | | tRNA | cytosol | tRNA-initiator Met(CAU) | | | 4.08 |
| aspV^a^ | b0216 | | tRNA | cytosol | tRNA-Asp(GUC) | | | 3.94 |
| argX^a^ | b3796 | | tRNA | cytosol | tRNA-Arg(CCG) | | | 3.81 |
| metY^a^ | b3171 | | tRNA | cytosol | tRNA-initiator Met(CAU) | | | 3.27 |
| serU^a^ | b1975 | | tRNA | cytosol | tRNA-Ser(CGA) | | | 3.02 |
| leuV | b4368 | | tRNA | cytosol | tRNA-Leu(CAG) | | | 2.86 |
| lysQ | b0749 | | tRNA | cytosol | tRNA-Lys(UUU) | | | 2.71 |
| ileV^a^ | b0202 | | tRNA | cytosol | tRNA-Ile(GAU) | | | 2.62 |
| serX | b1032 | | tRNA | cytosol | tRNA-Ser(GGA) | | | 2.55 |
| leuX^a^ | b4270 | | tRNA | cytosol | tRNA-Leu(CAA) | | | 2.32 |
| asnT | b1977 | | tRNA | cytosol | tRNA-Asn(GUU) | | | 2.30 |
| alaU^a^ | b3276 | | tRNA | cytosol | tRNA-Ala(UGC) | | | 2.28 |
| ileU^a^ | b3277 | | tRNA | cytosol | tRNA-Ile(GAU) | | | 2.26 |
| gltW | b2590 | | tRNA | cytosol | tRNA-Glu(UUC) | | | 2.22 |
| ileT^a^ | b3852 | | tRNA | cytosol | tRNA-Ile(GAU) | | | 2.18 |
| alaV | b0203 | | tRNA | cytosol | tRNA-Ala(UGC) | | | 2.13 |
| metW^a^ | b2815 | | tRNA | cytosol | tRNA-initiator Met(CAU) | | | 2.12 |
| thrV | b3273 | | tRNA | cytosol | tRNA-Thr(GGU) | | | 2.03 |
| thrW | b0244 | | tRNA | cytosol | tRNA-Thr(CGU) | | | 2.01 |
| glyW | b1911 | | tRNA | cytosol | tRNA-Gly(GCC) | | | 2.00 |
| **other** |  | |  |  |  | | |  |
| ffs^a^ | b0455 | | ncRNA | cytosol | signal recognition particle 4.5S RNA | | | 35.04 |
| yjcS | b4083 | | mRNA | periplasmic space | linear primary-alkylsulfatase | | | 8.79 |
| agaC | b3139 | | mRNA | inner membrane | galactosamine-specific PTS enzyme IIC component | | | 6.94 |
| sfmF^a^ | b0534 | | mRNA | extracellular space, pilus | putative fimbrial protein SfmF | | | 6.48 |
| insA-3^a^ | b0275 | | mRNA | cytosol | IS1 protein InsA | | | 5.19 |
| yghD | b2968 | | mRNA | inner membrane | putative type II secretion system M-type protein YghD | | | 3.61 |
| csgG^a^ | b1037 | | mRNA | periplasmic space, inner membrane, outer membrane | curli secretion channel | | | 3.56 |
| ftsL^a^ | b0083 | | mRNA | inner membrane | cell division protein FtsL | | | 3.35 |
| sdsN | b4719 | | ncRNA | no annotation | small regulatory RNA SdsN | | | 3.26 |
| elyC | b0920 | | mRNA | inner membrane | envelope biogenesis factor | | | 3.20 |
| ydcO | b1433 | | mRNA | inner membrane | putative transport protein YdcO | | | 3.02 |
| cutC^a^ | b1874 | | mRNA | cytosol | protein CutC | | | 3.00 |
| yiaC | b3550 | | mRNA | inner membrane, cytosol | peptidyl-lysineN-acetyltransferase YiaC | | | 2.83 |
| hybE^a^ | b2992 | | mRNA | cytosol | hydrogenase 2-specific chaperone | | | 2.77 |
| fsaA | b0825 | | mRNA | cytosol | fructose-6-phosphate aldolase 1 | | | 2.76 |
| gluQ^a^ | b0144 | | mRNA | cytosol | glutamyl-Q tRNAAspsynthetase | | | 2.69 |
| ygdG | b2798 | | mRNA | cytosol | flap endonuclease | | | 2.41 |
| trmO^a^ | b0195 | | mRNA | cytosol | tRNA m6t6A37 methyltransferase | | | 2.32 |
| rnpB | b3123 | | ncRNA | no annotation | RNase P catalytic RNA component | | | 2.31 |
| feoC | b3410 | | mRNA | cytosol | ferrous iron transport protein FeoC | | | 2.21 |
| smf^a^ | b4473 | | mRNA | cytosol | protein Smf | | | 2.20 |
| nlpC^a^ | b1708 | | mRNA | inner membrane, periplasmic space | NlpC/P60 family lipoprotein NlpC | | | 2.18 |
| nrfE | b4074 | | mRNA | inner membrane | putative cytochrome c-type biogenesis protein NrfE | | | 2.16 |
| yrfG | b3399 | | mRNA | cytosol | purine nucleotidase | | | 2.13 |
| hypD^a^ | b2729 | | mRNA | cytosol | Fe-(CN)2CO cofactor assembly scaffold protein HypD | | | 2.09 |
| rlmI | b0967 | | mRNA | cytosol | 23S rRNA m5C1962 methyltransferase | | | 2.06 |
| yadI | b0129 | | mRNA | cytosol, membrane | putative PTS enzyme IIA component YadI | | | 2.04 |
| rnd^a^ | b1804 | | mRNA | cytosol | RNase D | | | 2.02 |
| ssrS^a^ | b2911 | | ncRNA | no annotation | 6S RNA | | | 21.89 |
| ryjA^a^ | b4459 | | ncRNA | no annotation | small RNA RyjA | | | 11.73 |
| insA-2 | b0265 | | mRNA | cytosol | IS1 protein InsA | | | 11.64 |
| sroH^a^ | b4691 | | ncRNA | no annotation | small RNA SroH | | | 9.10 |
| ykgR^a^ | b4671 | | mRNA | inner membrane | uncharacterized membrane protein YkgR | | | 8.33 |
| ryeA^a^ | b4432 | | ncRNA | no annotation | small antisense RNA RyeA | | | 5.13 |
| ychQ^a^ | b1213 | | mRNA | inner membrane | SirB family protein YchQ | | | 4.09 |
| yfiM^a^ | b2586 | | mRNA | inner membrane | protein YfiM | | | 3.83 |
| ypdK^a^ | b4680 | | mRNA | inner membrane | uncharacterized membrane protein YpdK | | | 3.51 |
| yciY^a^ | b4595 | | mRNA | cytosol | uncharacterized protein YciY | | | 3.33 |
| yncL^a^ | b4598 | | mRNA | inner membrane | uncharacterized protein YncL | | | 3.26 |
| azuC^a^ | b4663 | | mRNA | inner membrane | uncharacterized protein AzuC | | | 3.14 |
| yohP^a^ | b4679 | | mRNA | inner membrane | uncharacterized membrane protein YohP | | | 3.14 |
| chiQ | b0682 | | mRNA | inner membrane, periplasmic space | lipoprotein ChiQ | | | 3.10 |
| ryjB^a^ | b4624 | | ncRNA | no annotation | small RNA RyjB | | | 2.97 |
| sraB^a^ | b4418 | | ncRNA | no annotation | small RNA SraB | | | 2.87 |
| ytfF^a^ | b4210 | | mRNA | inner membrane | inner membrane protein YtfF | | | 2.81 |
| ypeB^a^ | b4546 | | mRNA | cytosol | PF12843 family protein YpeB | | | 2.48 |
| yehM | b2120 | | mRNA | cytosol | uncharacterized protein YehM | | | 2.41 |
| yebB | b1862 | | mRNA | cytosol | putative papain-like amidase YebB | | | 2.40 |
| ycaR^a^ | b0917 | | mRNA | cytosol | PF03966 family protein YcaR | | | 2.39 |
| ybjO | b0858 | | mRNA | inner membrane | putative inner membrane protein | | | 2.38 |
| yecH^a^ | b1906 | | mRNA | cytosol | DUF2492 domain-containing protein YecH | | | 2.38 |
| yedL | b1932 | | mRNA | cytosol | putative acetyltransferase YedL | | | 2.38 |
| dsrB^a^ | b1952 | | mRNA | cytosol | protein DsrB | | | 2.34 |
| yniD^a^ | b4535 | | mRNA | inner membrane | uncharacterized protein YniD | | | 2.24 |
| yhaL | b3107 | | mRNA | cytosol | uncharacterized protein YhaL | | | 2.18 |
| fumD^a^ | b1675 | | mRNA | cytosol | fumarase D | | | 2.16 |
| yzfA | b4223 | | mRNA | no annotation | phantom gene yzfA | | | 2.12 |
| yecF^a^ | b1915 | | mRNA | cytosol | DUF2594 domain-containing protein YecF | | | 2.09 |
| tfaP^a^ | b1155 | | mRNA | cytosol | e14 prophage; putative tail fiber assembly protein TfaP | | | 2.05 |
