# Supplementary material for: Genome-wide identification of Kanamycin B binding RNA in Escherichia coli
Source: BMC Genomics. 2023 Mar 16;24:120. doi: 10.1186/s12864-023-09234-3 (PMC10018874; doi:10.1186/s12864-023-09234-3)
Supplement: Supplementary file 3 — Additional file 3: Table S3. 134 overlapped enrichment genes in 0 μM Kanamycin B and 1 μM Kanamycin B pull down assay. [file 12864_2023_9234_MOESM3_ESM.docx]

**Table S3. 134 overlapped enrichment genes in 0μM Kanamycin B and 1μM Kanamycin B pull down assay.**

| **Transcript Name** | **Transcript ID** | **Type** | **Location** | **Product** | **0μM pull down/total fold change** | **1μM pull down/total fold change** |  |
| --- | --- | --- | --- | --- | --- | --- | --- |
| **cell death** |  |  |  |  |  |  |  |
| ibsC | b4665 | mRNA | inner membrane | toxic peptide IbsC | 44.06 | 60.49 |  |
| ibsD | b4664 | mRNA | inner membrane | putative toxic peptide IbsD | 38.80 | 24.27 |  |
| ldrA | b4419 | mRNA | inner membrane | small toxic polypeptide LdrA | 3.64 | 5.02 |  |
| shoB | b4687 | mRNA | inner membrane | toxic peptide ShoB | 3.35 | 4.92 |  |
| ldrC | b4423 | mRNA | inner membrane | small toxic polypeptide LdrC | 3.95 | 3.23 |  |
| **post-transcriptional gene silencing by RNA** | | | |  |  |  |  |
| sibD | b4447 | ncRNA | no annotation | ncRNA | 9.92 | 14.08 |  |
| sibC | b4446 | ncRNA | no annotation | ncRNA | 4.70 | 5.04 |  |
| sibE | b4611 | ncRNA | no annotation | ncRNA | 3.64 | 4.90 |  |
| sibB | b4437 | ncRNA | no annotation | ncRNA | 3.44 | 4.34 |  |
| sibA | b4436 | ncRNA | no annotation | ncRNA | 3.27 | 2.52 |  |
| **transcription, DNA-templated** | | |  |  |  |  |  |
| lrhA | b2289 | mRNA | cytosol | DNA-binding transcriptional dual regulator LrhA | 7.25 | 6.44 |  |
| pyrL | b4246 | mRNA | cytosol | pyrBI operon leader peptide | 3.35 | 3.59 |  |
| cecR | b0796 | mRNA | cytosol | DNA-binding transcriptional dual regulator CecR | 2.49 | 2.21 |  |
| thrL | b0001 | mRNA | cytosol | thr operon leader peptide | 2.09 | 2.14 |  |
| hupB | b0440 | mRNA | cytosol | DNA-binding protein HU-beta | 2.29 | 2.07 |  |
| **response to stimulus** | | |  |  |  |  |  |
| glmY | b4441 | ncRNA | no annotation | ncRNA | 14.62 | 17.82 |  |
| ynhF | b4602 | mRNA | inner membrane | stress response membrane protein YnhF | 10.12 | 10.05 |  |
| ftsI | b0084 | mRNA | inner membrane | peptidoglycan DD-transpeptidase FtsI | 5.69 | 4.22 |  |
| acrZ | b0762 | mRNA | cytosol, outer membrane, inner membrane | multidrug efflux pump accessory protein AcrZ | 2.86 | 3.68 |  |
| allR | b0506 | mRNA | cytosol | DNA-binding transcriptional repressor AllR | 2.45 | 3.27 |  |
| rmf | b0953 | mRNA | cytosol | ribosome modulation factor | 2.92 | 3.20 |  |
| dinI | b1061 | mRNA | cytosol | DNA damage-inducible protein I | 2.84 | 3.20 |  |
| dinQ | b4613 | mRNA | inner membrane | UV inducible membrane toxin DinQ | 4.02 | 3.11 |  |
| ybfA | b0699 | mRNA | inner membrane, cytosol | DUF2517 domain-containing protein YbfA | 2.78 | 2.97 |  |
| ecnB | b4411 | mRNA | inner membrane, outer membrane | bacteriolytic entericidin B lipoprotein | 3.20 | 2.85 |  |
| mgtS | b4599 | mRNA | inner membrane | small protein MgtS | 3.25 | 2.78 |  |
| yqaE | b2666 | mRNA | inner membrane | Pmp3 family protein | 4.41 | 2.72 |  |
| yphB | b2544 | mRNA | cytosol | putative aldose 1-epimerase YphB | 3.57 | 2.33 |  |
| mgtL | b4702 | mRNA | no annotation | leader peptide MgtL | 2.10 | 2.24 |  |
| oxyS | b4458 | ncRNA | bacterial nucleoid, cytosol | ncRNA | 3.74 | 2.15 |  |
| sulA | b0958 | mRNA | inner membrane | cell division inhibitor SulA | 2.16 | 2.14 |  |
| ytfK | b4217 | mRNA | no annotation | DUF1107 domain-containing protein YtfK | 2.06 | 2.05 |  |
| **biosynthetic process** | | |  |  |  |  |  |
| hisL | b2018 | mRNA | cytosol | his operon leader peptide | 5.89 | 7.99 |  |
| ilvL | b3766 | mRNA | cytosol | ilvXGMEDA operon leader peptide | 5.46 | 4.26 |  |
| leuL | b0075 | mRNA | cytosol | leu operon leader peptide | 2.97 | 3.86 |  |
| cof | b0446 | mRNA | cytosol | HMP-PP phosphatase | 2.63 | 2.88 |  |
| leuD | b0071 | mRNA | cytosol | 3-isopropylmalate dehydratase subunit LeuD | 3.01 | 2.70 |  |
| yhbV | b3159 | mRNA | no annotation | putative peptidase YhbV | 3.84 | 2.69 |  |
| coaD | b3634 | mRNA | cytosol | pantetheine-phosphate adenylyltransferase | 2.58 | 2.15 |  |
| **catabolic process** | |  |  |  |  |  |  |
| allB | b0512 | mRNA | cytosol | allantoinase | 3.81 | 3.12 |  |
| dgoD | b4478 | mRNA | no annotation | D-galactonate dehydratase | 2.98 | 3.10 |  |
| fucI | b2802 | mRNA | cytosol | L-fucose isomerase | 2.54 | 2.70 |  |
| chiA | b3338 | mRNA | extracellular space, periplasmic space | endochitinase | 2.50 | 2.05 |  |
| **regulation of single-species biofilm formation** | | | | |  |  |  |
| ryfD | b4609 | ncRNA | no annotation | ncRNA | 32.24 | 45.91 |  |
| dsrA | b1954 | ncRNA | no annotation | ncRNA | 4.42 | 3.98 |  |
| csrC | b4457 | ncRNA | no annotation | ncRNA | 2.97 | 2.13 |  |
| **lipid metabolic process** | | |  |  |  |  |  |
| lpp | b1677 | mRNA | outer membrane, cell wall, extracellular space, periplasmic space | murein lipoprotein | 2.20 | 2.57 |  |
| lapA | b1279 | mRNA | inner membrane | lipopolysaccharide assembly protein A | 2.26 | 2.31 |  |
| **DNA recombination** | |  |  |  |  |  |  |
| insQ | b1432 | mRNA | no annotation | putative insertion element transposase InsQ | 2.33 | 2.39 |  |
| sbcD | b0398 | mRNA | cytosol | ATP dependent, structure specific DNA nuclease - SbcD subunit | 2.19 | 2.22 |  |
| fimE | b4313 | mRNA | cytosol | regulator for fimA | 2.92 | 2.08 |  |
| **translation** | |  |  |  |  |  |  |
| fnrS | b4699 | ncRNA | no annotation | ncRNA | 11.35 | 11.32 |  |
| glmZ | b4456 | ncRNA | no annotation | ncRNA | 7.00 | 7.34 |  |
| spf | b3864 | ncRNA | no annotation | ncRNA | 3.72 | 4.43 |  |
| omrB | b4445 | ncRNA | no annotation | ncRNA | 3.71 | 4.27 |  |
| gcvB | b4443 | ncRNA | no annotation | ncRNA | 3.09 | 3.87 |  |
| istR | b4616 | ncRNA | no annotation | ncRNA | 6.29 | 3.37 |  |
| mgrR | b4698 | ncRNA | no annotation | ncRNA | 3.12 | 2.09 |  |
| **transport** |  |  |  |  |  |  |  |
| fieF | b3915 | mRNA | inner membrane | Zn(2(+))/Fe(2(+))/Cd(2(+)) exporter | 2.29 | 3.13 |  |
| araE | b2841 | mRNA | inner membrane | arabinose:H(+) symporter | 2.86 | 2.69 |  |
| xylG | b3567 | mRNA | inner membrane | xylose ABC transporter ATP binding subunit | 4.53 | 2.35 |  |
| ycaD | b0898 | mRNA | inner membrane | putative transporter YcaD | 2.66 | 2.25 |  |
| dauA | b1206 | mRNA | inner membrane | aerobic C4-dicarboxylate transporter DauA | 2.43 | 2.24 |  |
| eamB | b2578 | mRNA | inner membrane | cysteine/O-acetylserine exporter EamB | 2.08 | 2.22 |  |
| yfeO | b2389 | mRNA | inner membrane | putative transport protein YfeO | 2.19 | 2.08 |  |
| **rRNA** |  |  |  |  |  |  |  |
| rrfF | b3272 | rRNA | cytosol | 5S ribosomal RNA | 46.07 | 61.87 |  |
| rrfB | b3971 | rRNA | cytosol | 5S ribosomal RNA | 44.28 | 56.83 |  |
| rrfE | b4010 | rRNA | cytosol | 5S ribosomal RNA | 42.11 | 56.12 |  |
| rrfG | b2588 | rRNA | cytosol | 5S ribosomal RNA | 36.00 | 48.15 |  |
| rrfD | b3274 | rRNA | cytosol | 5S ribosomal RNA | 34.85 | 45.71 |  |
| rrfH | b0205 | rRNA | cytosol | 5S ribosomal RNA | 35.27 | 45.14 |  |
| rrfC | b3759 | rRNA | cytosol | 5S ribosomal RNA | 30.58 | 38.82 |  |
| rrfA | b3855 | rRNA | no annotation | 5S ribosomal RNA | 12.07 | 18.27 |  |
| **tRNA** |  |  |  |  |  |  |  |
| trpT | b3761 | tRNA | cytosol | tRNA | 13.02 | 49.42 |  |
| alaW | b2397 | tRNA | cytosol | tRNA | 10.81 | 15.94 |  |
| selC | b3658 | tRNA | cytosol | tRNA | 5.52 | 9.63 |  |
| valU | b2401 | tRNA | cytosol | tRNA | 6.92 | 8.60 |  |
| leuU | b3174 | tRNA | cytosol | tRNA | 12.13 | 7.92 |  |
| aspU | b0206 | tRNA | cytosol | tRNA | 3.85 | 7.34 |  |
| gltU | b3757 | tRNA | cytosol | tRNA | 4.83 | 6.94 |  |
| leuZ | b1909 | tRNA | cytosol | tRNA | 3.80 | 6.19 |  |
| glyT | b3978 | tRNA | cytosol | tRNA | 10.74 | 5.97 |  |
| leuQ | b4370 | tRNA | cytosol | tRNA | 5.11 | 5.23 |  |
| gltV | b4008 | tRNA | cytosol | tRNA | 3.87 | 4.92 |  |
| metV | b2816 | tRNA | cytosol | tRNA | 2.85 | 4.62 |  |
| serV | b2695 | tRNA | cytosol | tRNA | 3.58 | 4.29 |  |
| metZ | b2814 | tRNA | cytosol | tRNA | 4.49 | 4.08 |  |
| aspV | b0216 | tRNA | cytosol | tRNA | 3.52 | 3.94 |  |
| argX | b3796 | tRNA | cytosol | tRNA | 3.68 | 3.81 |  |
| metY | b3171 | tRNA | cytosol | tRNA | 3.11 | 3.27 |  |
| serU | b1975 | tRNA | cytosol | tRNA | 4.48 | 3.02 |  |
| ileV | b0202 | tRNA | cytosol | tRNA | 3.80 | 2.62 |  |
| leuX | b4270 | tRNA | cytosol | tRNA | 4.11 | 2.32 |  |
| alaU | b3276 | tRNA | cytosol | tRNA | 2.21 | 2.28 |  |
| ileU | b3277 | tRNA | cytosol | tRNA | 3.49 | 2.26 |  |
| ileT | b3852 | tRNA | cytosol | tRNA | 3.20 | 2.18 |  |
| metW | b2815 | tRNA | cytosol | tRNA | 2.27 | 2.12 |  |
| **other** |  |  |  |  |  |  |  |
| ffs | b0455 | ncRNA | cytosol | ncRNA | 17.33 | 35.04 |  |
| sfmF | b0534 | mRNA | extracellular space, pilus | putative fimbrial protein SfmF | 3.95 | 6.48 |  |
| insA-3 | b0275 | mRNA | no annotation | IS1 protein InsA | 3.62 | 5.19 |  |
| csgG | b1037 | mRNA | periplasmic space, inner membrane, outer membrane | curli secretion channel | 4.09 | 3.56 |  |
| ftsL | b0083 | mRNA | inner membrane | cell division protein FtsL | 2.28 | 3.35 |  |
| cutC | b1874 | mRNA | cytosol | protein CutC | 2.97 | 3.00 |  |
| ygfS | b2886 | mRNA | no annotation | putative oxidoreductase, 4Fe-4S ferredoxin-type subunit | 2.85 | 2.93 |  |
| hybE | b2992 | mRNA | no annotation | hydrogenase 2-specific chaperone | 2.16 | 2.77 |  |
| gluQ | b0144 | mRNA | cytosol | glutamyl-Q tRNA(Asp) synthetase | 2.39 | 2.69 |  |
| trmO | b0195 | mRNA | cytosol | tRNA m(6)t(6)A37 methyltransferase | 4.50 | 2.32 |  |
| smf | b4473 | mRNA | cytosol | protein Smf | 2.16 | 2.20 |  |
| nlpC | b1708 | mRNA | inner membrane, periplasmic space | NlpC/P60 family lipoprotein NlpC | 2.33 | 2.18 |  |
| hypD | b2729 | mRNA | cytosol | Fe-(CN)2CO cofactor assembly scaffold protein HypD | 2.09 | 2.09 |  |
| rnd | b1804 | mRNA | cytosol | RNase D | 2.22 | 2.02 |  |
| ssrS | b2911 | ncRNA | no annotation | ncRNA | 9.97 | 21.89 |  |
| ryjA | b4459 | ncRNA | no annotation | ncRNA | 12.34 | 11.73 |  |
| sroH | b4691 | ncRNA | no annotation | ncRNA | 5.55 | 9.10 |  |
| ykgR | b4671 | mRNA | inner membrane | putative membrane protein YkgR | 7.04 | 8.33 |  |
| ryeA | b4432 | ncRNA | no annotation | ncRNA | 5.26 | 5.13 |  |
| ychQ | b1213 | mRNA | inner membrane | SirB family protein YchQ | 7.25 | 4.09 |  |
| yfiM | b2586 | mRNA | no annotation | protein YfiM | 3.94 | 3.83 |  |
| ypdK | b4680 | mRNA | inner membrane | putative membrane protein YpdK | 3.83 | 3.51 |  |
| yciY | b4595 | mRNA | no annotation | uncharacterized protein YciY | 2.89 | 3.33 |  |
| yncL | b4598 | mRNA | inner membrane | uncharacterized protein YncL | 4.16 | 3.26 |  |
| azuC | b4663 | mRNA | inner membrane | uncharacterized protein AzuC | 2.09 | 3.14 |  |
| yohP | b4679 | mRNA | inner membrane | putative membrane protein YohP | 3.57 | 3.14 |  |
| ryjB | b4624 | ncRNA | no annotation | ncRNA | 3.02 | 2.97 |  |
| sraB | b4418 | ncRNA | no annotation | ncRNA | 2.88 | 2.87 |  |
| ytfF | b4210 | mRNA | inner membrane | inner membrane protein YtfF | 2.46 | 2.81 |  |
| yebB | b1862 | mRNA | cytosol | putative papain-like amidase YebB | 2.75 | 2.40 |  |
| ycaR | b0917 | mRNA | cytosol | UPF0434 family protein YcaR | 2.46 | 2.39 |  |
| yecH | b1906 | mRNA | no annotation | DUF2492 domain-containing protein YecH | 2.96 | 2.38 |  |
| dsrB | b1952 | mRNA | cytosol | protein DsrB | 2.34 | 2.34 |  |
| yniD | b4535 | mRNA | inner membrane | uncharacterized protein YniD | 2.29 | 2.24 |  |
| fumD | b1675 | mRNA | cytosol | fumarase D | 2.28 | 2.16 |  |
| yecF | b1915 | mRNA | no annotation | DUF2594 domain-containing protein YecF | 2.10 | 2.09 |  |
| tfaP | b1155 | mRNA | cytosol | e14 prophage; putative tail fiber assembly protein TfaP | 2.23 | 2.05 |  |
