# Supplementary figures and images for: Genome-wide identification of Kanamycin B binding RNA in Escherichia coli
Source: BMC Genomics. 2023 Mar 16;24:120. doi: 10.1186/s12864-023-09234-3 (PMC10018874; doi:10.1186/s12864-023-09234-3)

# Additional file 4\_Fig. S1

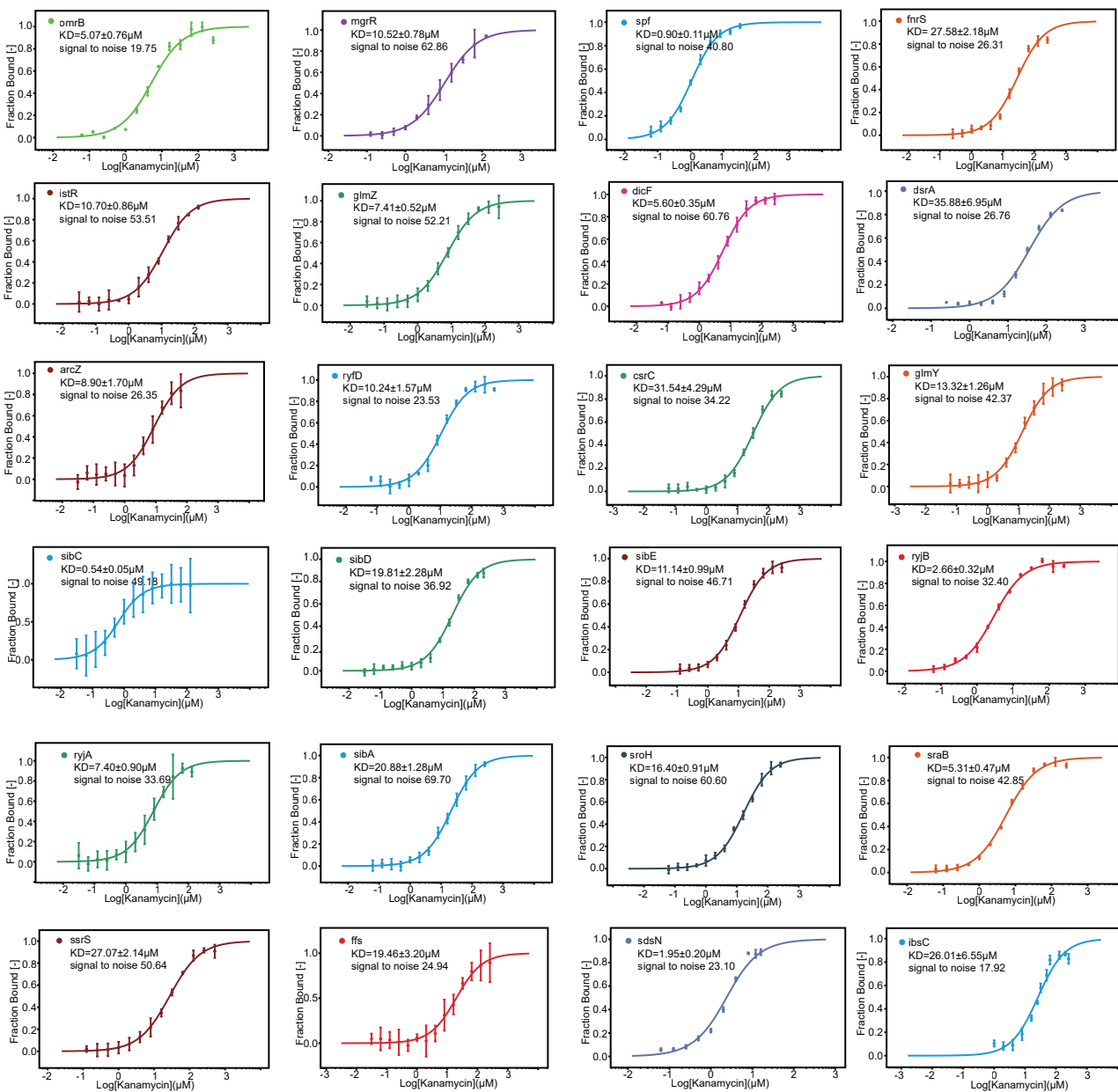

Supplement: Supplementary file 4 — Additional file 4: Fig. S1. Binding curve generated by MST for binding of Kanamycin B to 24 enriched RNA with Kanamycin B measured by MST. [file 12864_2023_9234_MOESM4_ESM.pdf]

Additional file 5\_Fig. S2

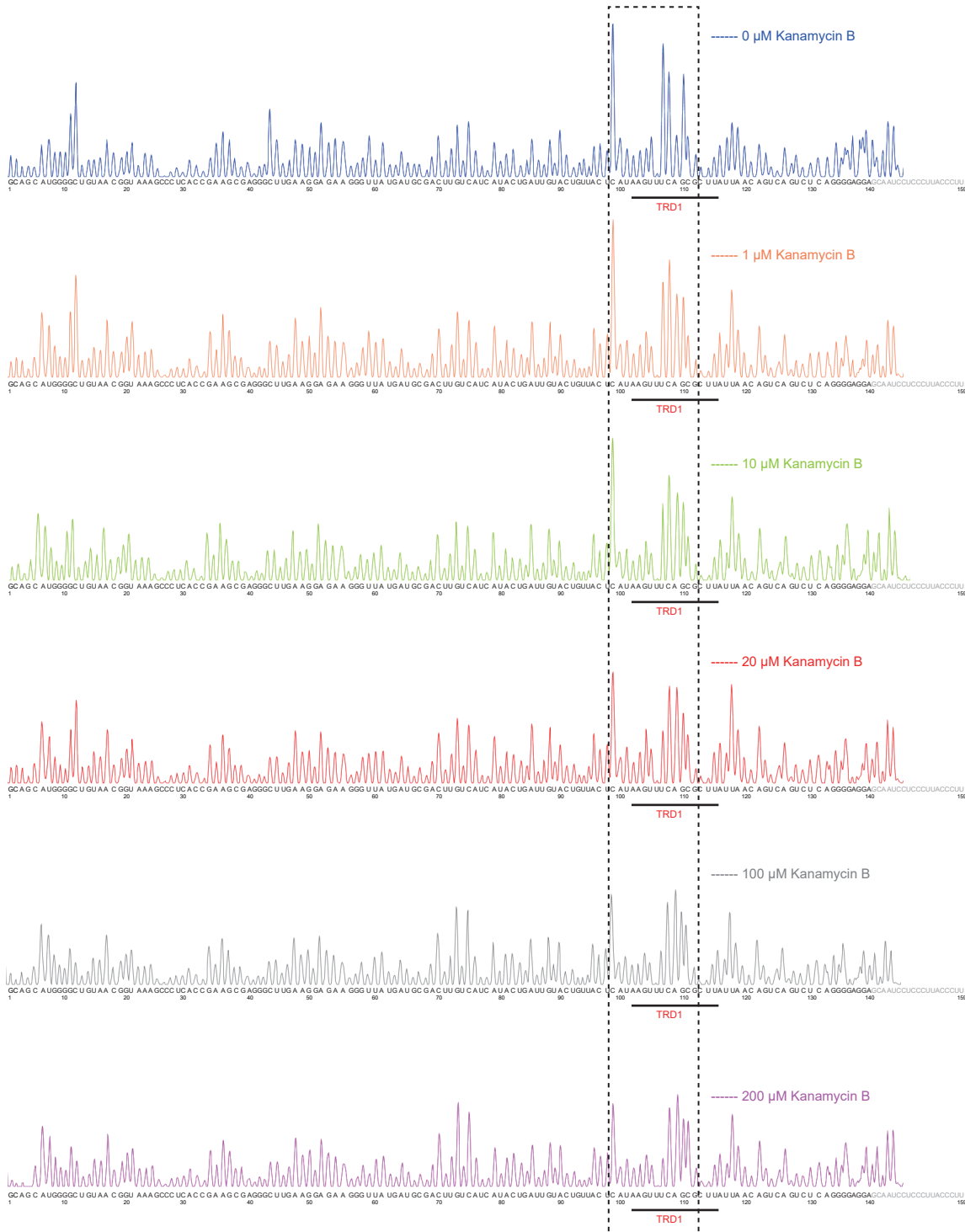

Supplement: Supplementary file 5 — Additional file 5: Fig. S2. Electropherogram of NMIA-modification on ibsC RNA on Kanamycin B titration. [file 12864_2023_9234_MOESM5_ESM.pdf]

Additional file 6\_Fig. S3

**a**

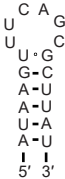

ibsC

**b**

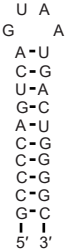

spf

**c**

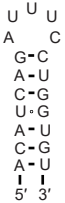

dsrA

**d**

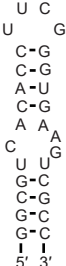

16s rRNA A-site

Supplement: Supplementary file 6 — Additional file 6: Fig. S3. Comparison of a secondary structure of RNA against Kanamycin B. a stem loop of ibsC. b stem loop of spf. c stem loop of dsrA. d stem loop of the 16 s rRNA A-site model. [file 12864_2023_9234_MOESM6_ESM.pdf]
